# An integrated approach of gene expression and DNA-methylation profiles of WNT signaling genes uncovers novel prognostic markers in Acute Myeloid Leukemia

**DOI:** 10.1186/1471-2105-16-S4-S4

**Published:** 2015-02-23

**Authors:** Erdogan Taskesen, Frank JT Staal, Marcel JT Reinders

**Affiliations:** 1 Delft Bioinformatics Lab (DBL), Delft University of Technology, 2628CD Delft, the Netherlands; 2 Netherlands Bioinformatics Centre (NBIC), the Netherlands; 3 Department of Immunohematology and Bloodtransfusion, 2300RC Leiden, the Netherlands

**Keywords:** Data integration, Gene expression, DNA-methylation, Prognostic markers, Acute Myeloid Leukemia, Wingless-Int (WNT)

## Abstract

**Background:**

The wingless-Int (WNT) pathway has an essential role in cell regulation of hematopoietic stem cells (HSC). For Acute Myeloid Leukemia (AML), the malignant counterpart of HSC, currently only a selective number of genes of the WNT pathway are analyzed by using either gene expression or DNA-methylation profiles for the identification of prognostic markers and potential candidate targets for drug therapy. It is known that mRNA expression is controlled by DNA-methylation and that specific patterns can infer the ability to differentiate biological differences, thus a combined analysis using all WNT annotated genes could provide more insight in the WNT signaling.

**Approach:**

We created a computational approach that integrates gene expression and DNA promoter methylation profiles. The approach represents the continuous gene expression and promoter methylation profiles with nine discrete mutually exclusive scenarios. The scenario representation allows for a refinement of patient groups by a more powerful statistical analysis, and the construction of a co-expression network. We focused on 268 WNT annotated signaling genes that are derived from the molecular signature database.

**Results:**

Using the scenarios we identified seven prognostic markers for overall survival and event-free survival. Three genes are novel prognostic markers; two with favorable outcome (*PSMD2, PPARD*) and one with unfavorable outcome (*XPNPEP*). The remaining four genes (*LEF1, SFRP2, RUNX1*, and *AXIN2*) were previously identified but we could refine the patient groups. Three AML risk groups were further analyzed and the co-expression network showed that only the good risk group harbors frequent promoter hypermethylation and significantly correlated interactions with proteasome family members.

**Conclusion:**

Our results provide novel insights in WNT signaling in AML, we discovered new and previously identified prognostic markers and a refinement of the patient groups.

## Background

The development of stem cells and progenitor cells into mature functioning cells depends on, among others, the correct functioning of the WNT signaling pathway [1,2]. As an example, Wnt3a deficiency leads in leukemia cells to reduced numbers of long-term hematopoietic stem cells (HSC) and multipotent progenitors [3,4]. In Acute Myeloid Leukemia (AML), the WNT pathway genes do not show frequent genetic alterations but rather gene silencing by promoter methylation [5-7]. The epigenetic alterations plays a critical role in initiation, progression, and maintenance of the disease phenotype. Promoter methylation of genes in the WNT pathway (e.g., *AXIN2, sFRP1, sFRP2, sFRP4, sFRP5, DKK1, DKK3, APC, RUNX1, SOX17, WIF1, RASSF1A, LKB1/STK11, cyclin D1, TCF1, LEF1. CTNNB1*) causes genes to be downregulated in their mRNA expression, thereby deregulation of the WNT signaling. The relation between these genetic and epigenetic factors is especially important when studying the disease phenotype, such as for patients with deregulated WNT signaling. This is also shown in previous studies that reported associations of promoter methylation of WNT signaling genes with adverse prognosis [6,8].

Over the past years, genetic and epigenetic studies have, independently from each other, provided important new insights in the underlying pathology of Acute Myeloid Leukemia (AML) [9-11]. AML is a heterogeneous disease that can roughly be classified in three risk groups; good, intermediate and the poor risk group. The good risk group consists of patients with a translocation between chromosome 15 and 17 (t(15;17)) or a translocation between chromosomes 8 and 21 (t(8;21) or an inversion of chromosome 16 (inv(16)). The intermediate risk group consists patients with a normal karyotype, and the poor risk group with a complex karyotype (patients more than 3 cytogenetic abnormalities). Previous studies analysed genetic and epigenetic changes of WNT signaling genes mostly in patients with intermediate risk [6], however, DNA hypermethylation was found to be enriched for the good risk group [12]. These studies however analysed a limited number of genes in the WNT signaling pathway. A comprehensive analysis of the relationships between DNA-methylation and transcript expression by means of all annotated genes of the WNT signaling pathway has not been done.

For this study we developed a statistical approach to model the mRNA transcript expression with DNA-methylation abundance and applied it to a complete list of WNT signaling genes. This allowed us to refine groups of patients from which genes have specific promoter methylation (either hypermethylated, hypomethylated or not methylated at all), and gene expression (either up regulated, down regulated or not changed in expression), potentially important for better clinical diagnostics. Further we provided novel insights of WNT signaling in AML that may inspire therapeutic interventions. Our integration strategy provides useful biological interpretation for the detected prognostic markers and for patient clustering.

## Results

### Analysis of prognostic markers by means of gene-state scenarios

**Figure 1:**
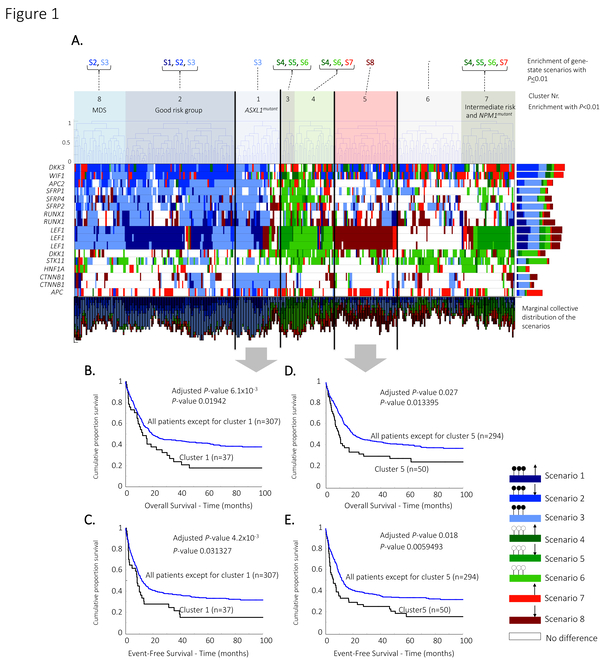
**Hierarchical clustering by means of the scenarios, and Kaplan-Meier curves**. Leukemia related WNT signaling genes (13 genes, 17 refseqs) are classified in the mutually exclusive scenarios, and (A) hierarchically clustered using hamming distance and complete linkage. The dendrogram is cut at the top level which resulted in eight distinctive clusters. Significantly enriched scenarios, cytogenetical characteristics and molecular abnormalities are described. (B) Patients in cluster 1, and cluster 5 show significant unfavourable outcome in overall survival, and (C) event-free survival in a univariate analysis (log-rank test), and after correcting for known prognostic markers in a multivariate analysis. The horizontal and vertical bar plots illustrates the marginal collective distribution of the scenarios among the genes, and samples respectively.

It has previously been shown that DNA-methylation or gene expression levels of genes can be used as predictive features to determine clinical outcome of AML patients [6,8]. We speculated that patient groups can be refined when DNA-methylation and gene expression levels of genes are analysed in an integrated fashion. We therefore categorized each gene in one of nine scenarios, each representing a unique combination of up/down/no gene expression and hyper/hypo/no methylation of its promoter region (see Methods and Figure 1A). We next assessed whether a state of a gene is predictive for clinical outcome for Overall Survival (OS) and Event-Free Survival (EFS) using univariate (log-rank test) and multivariate analyses (Cox's proportional hazard ratio model) (See method section). We used in total 344 AML patients and marked a gene as a prognostic marker if both the univariate and multivariate analysis resulted in significant differences in clinical outcome with P ≤ 0.05 (See method section and Additional file 1).

### Re-analysis of known WNT associated prognostic markers for leukemia

We started analysing thirteen WNT signaling genes that are known to have important function in leukemia, i.e., *sFRP1, sFRP2, sFRP4, DKK1, DKK3, APC, APC2, RUNX1, WIF1, TCF1, LEF1, LKB1/STK11*, and *CTNNB1*, and could confirm on our data that *LEF1 *[13], *SFRP2 *[6], *RUNX1 *[14], and *AXIN2 *have prognostic value.

#### *AXIN2*

**Figure 2:**
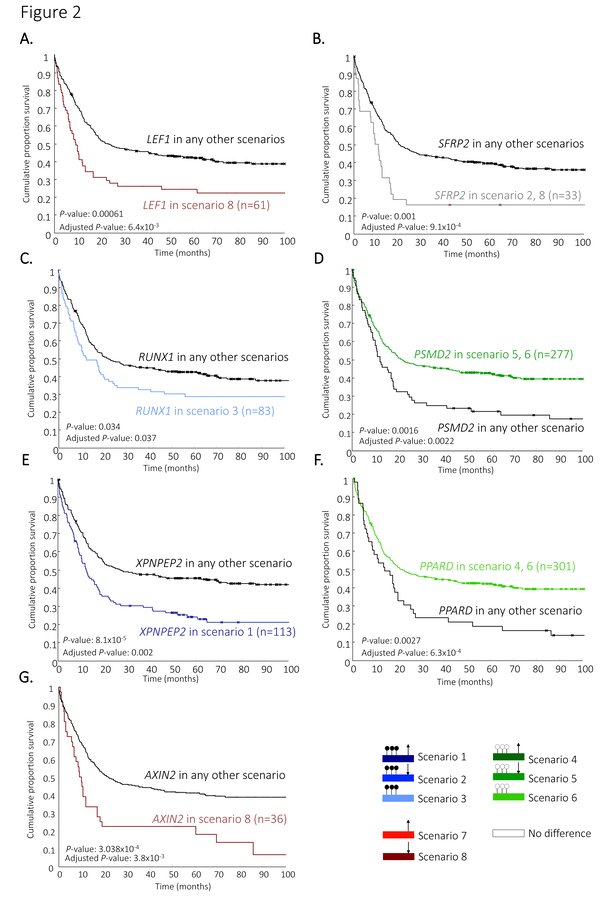
**Kaplan-Meier curves for the identified prognostic genes**. The Kaplan-Meier curves for overall survival illustrates the identified prognostic markers and the patient groups based on the classification of gene scenario. (A) *LEF1*, (B) *SFRP2*, (C) *RUNX1*, (D) *PSMD2*, (E) *XPNPEP2*, (F) *PPARD*, and (G) *AXIN2*. Significance for each prognostic marker is computed by comparing patients in the particular scenario versus patients in any other scenario (black line) using a univariate (log-rank test, indicated by the *P*-value), and multivariate analysis (Cox's proportional hazard ratio model, indicated by the adjusted *P*-value).

*AXIN *inhibition protein 2 (*AXIN2*) is a well-established readout for WNT signaling activation across various species and has been used within the hematopoietic system in reporter mice as readout of WNT activation[15]. We detected that the expression levels of *AXIN2 *separates patients in two groups for which unfavourable outcome is seen for patients with significant downregulated *AXIN2 *expression levels in OS (Figure 2G, Table 1) and EFS (Additional file 2G, and 3). Note that this is irrespective of the DNA-methylation levels in the promoter region of *AXIN2 *(i.e., *AXIN2*'s genes-state is in scenario 8). Surprisingly, this is in contrast to solid tumours, for instance colon carcinomas, in which high WNT signaling is a risk factor.

**Table 1:** Multivariate analysis for the identified prognostic genes for overall survival (OS).

Variables	*P-*value	HR	95% CI-low	CI-high
**Overall survival**				
*LEF1 *(scenario 8)	0.00642	1.58	1.14	2.19
*CEBPA*^dmα^	0.00214	0.33	0.16	0.67
*FLT3^ITD β^*	0.00025	1.77	1.30	2.40
*NPM1^+β^*	0.00005	0.51	0.37	0.71
WBC count^δ^, × 10^9^/L	0.00886	1.00	1.00	1.00
Age^$^	0.00005	1.02	1.01	1.03

**Overall survival**				
*SFRP2 *(scenario 2, 8)	0.00091	2.00	1.33	3.00
*CEBPA*^dmα^	0.00160	0.32	0.16	0.65
*FLT3^ITD β^*	0.00007	1.87	1.37	2.55
*NPM1^+ β^*	0.00006	0.52	0.38	0.71
WBC count^δ^, × 10^9^/L	0.00470	1.00	1.00	1.01
Age^$^	0.00007	1.02	1.01	1.03

**Overall survival**				
*RUNX1 *(scenario 3)	0.03768	1.38	1.02	1.88
*CEBPA*^dmα^	0.00308	0.34	0.17	0.69
*FLT3^ITD β^*	0.00023	1.78	1.31	2.41
*NPM1^+ β^*	0.00004	0.51	0.37	0.71
WBC count^δ^, × 10^9^/L	0.01106	1.00	1.00	1.00
Age^$^	0.00001	1.02	1.01	1.03

**Overall survival**				
*PSMD2 *(scenario 5, 6)	0.00220	0.60	0.43	0.83
*CEBPA*^dmα^	0.00130	0.31	0.15	0.63
*FLT3^ITD β^*	0.00006	1.87	1.38	2.55
*NPM1^+ β^*	0.00160	0.59	0.42	0.82
WBC count^δ^, × 10^9^/L	0.00270	1.00	1.00	1.01
Age^$^	0.00009	1.02	1.01	1.03

**Overall survival**				
*XPNPEP *(scenario 1)	0.00200	1.56	1.18	2.07
*CEBPA*^dmα^	0.00084	0.30	0.15	0.60
*FLT3^ITD β^*	0.00130	1.66	1.22	2.26
*NPM1^+ β^*	0.00020	0.55	0.40	0.75
WBC count^δ^, × 10^9^/L	0.01190	1.00	1.00	1.00
Age^$^	0.00015	1.02	1.01	1.03

**Overall survival**				
*PPARD *(scenario 4, 6)	0.00063	0.52	0.36	0.76
*CEBPA*^dmα^	0.00075	0.29	0.14	0.60
*FLT3^ITD β^*	0.00011	1.83	1.35	2.48
*NPM1^+ β^*	0.00084	0.57	0.41	0.79
WBC count^δ^, × 10^9^/L	0.00360	1.00	1.00	1.01
Age^$^	0.00001	1.02	1.01	1.03

**Overall survival**				
*AXIN2 *(scenario 8)	0.0038	1.79	1.21	2.66
*CEBPA*^dmα^	0.0009	0.30	0.15	0.61
*FLT3^ITD β^*	0.0003	1.77	1.30	2.40
*NPM1^+ β^*	0.0003	0.55	0.40	0.76
WBC count^δ^, × 10^9^/L	0.0100	1.00	1.00	1.00
Age^$^	0.00004	1.02	1.01	1.03

Cox proportional hazard model for multivariable analyses of prognostic markers for overall survival. Analyses included 344 AML patients. Abbreviations: HR, hazard ratio; CI, confidence interval; *α CEBPA^double−mutation ^*status versus *CEBPA^wt^*, *β FLT3^ITD ^*versus no *FLT3^ITD ^*mutation, *β NPM1^mutant ^*versus *NPM1^wt^*, *δ *WBC count higher than 20 × 10^9^/*L *versus lower than 20 × 10^9^/*L*, $ Age is used as continuous variable.

#### *LEF1*

For lymphoid enhancer-binding factor-1 (*LEF1*) it was shown that high gene expression levels are associated with favourable clinical outcome in CN-AML [13], and thus that downregulated expression levels are then associated with unfavourable clinical outcome. We indeed detected in our data that patients with significant downregulated *LEF1 *mRNA expression levels have an unfavourable clinical outcome for OS (Figure 2A, Table 1), and EFS (Additional file 2A, and 3). In addition, the unfavourable clinical outcome was only seen for patients that showed no significant differences of promoter DNA-methylation of *LEF1 *(i.e., patients with the gene-state of *LEF1 *in scenario 8). In fact, from the 97 patients with significant downregulated gene expression levels of *LEF1*, only 61 patients with *LEF1 *in scenario 8 could be marked with significant unfavourable clinical outcome whereas the other 36 patients (i.e., *LEF1 *in scenario 5) could not.

#### *SFRP2*

For the secreted frizzled-related protein 2 (*SFRP2*) it has previously been shown that DNA hypermethylation of the promoter region is associated with unfavourable clinical outcome [6]. In our data, we also detected significant unfavourable clinical outcome for patients with promoter hypermethylated *SFRP2 *(Figure 2B) but these patients also showed downregulated expression levels of *SFRP2 *(i.e., patients with *SFRP2 *in scenario 2). In addition to that, unfavourable clinical outcome was also seen for patients with solely downregulated expression levels of *SFRP2 *(i.e., patients with *SFRP2 *in scenario 8). These two patients groups were therefore combined in our approach, as described in the method section (OS: Figure 2B, Table 1 and EFS: Additional file 2B, and 3).

#### *RUNX1*

Runt-Related Transcription Factor is a tumor suppressor in myeloid neoplasms, and mutations in *RUNX1 *are previously associated with unfavourable outcome in CN-AMLs [14,16]. In addition, it was previously demonstrated that inhibition of *RUNX1 *activity could be a promising therapeutic strategy for AMLs with leukemogenic fusion proteins [17].We detected significant unfavourable clinical outcome in OS (Figure 2C, Table 1), but not for EFS (Additional file 2C, and 3), for patients with DNA hypermethylation but without significant differences of gene expression levels of *RUNX1 *(i.e., patients with *RUNX1 *in scenario 3). Thus we show that not all patients with DNA hypermethylation have poor survival; it also depends on the gene expression levels.

Taken together, by re-analysing known WNT associated markers, we could re-establish the prognostic role of a number of these markers in AML patients, but we also illustrate that patient groups could be refined by using an integrative analysis of gene expression and DNA-methylation profiles. A comparison of our results with the use of only gene expression profiles, we detected that only *SFRP2 *showed significant associations with OS and EFS, i.e., downregulated expression levels (scenario 2, 5 and 8) are associated with unfavourable clinical outcome compared to the upregulated expression levels (scenario 1, 4 and 7, additional file 4A, B). The use of solely DNA-methylation profiles did not result in significant associations with clinical outcome, i.e., hypermethylation (scenario 1, 2 and 3) versus hypomethylation (scenario 4, 5, 6).

### Detection of novel WNT associated prognostic markers for leukemia

In addition to the WNT signaling genes that are known to have important functions in leukemia, we also analysed the 268 WNT signaling genes annotated in the molecular signature database (see Methods). We speculated that novel prognostic markers could be identified by using the refined gene-state scenarios. Three additional genes (*PSMD2, XPNPEP2, and PPARD*) showed significant differences in OS and EFS.

#### *PSMD2*

The proteasome non-catalytic subunit is previously identified as a potential therapeutic target in lung adenocarcinomas [18]. For *PSMD2 *we detected significant favourable OS (Figure 2D, Table 1) and EFS (Additional file 2D, and 3), for patients with a promoter hypomethylation and downregulated gene expression levels (i.e., patients with the *PSMD2 *gene-state in scenario 5). In addition, favourable clinical outcome was also seen for patients with solely promoter hypomethylation (i.e., patients with the *PSMD2 *gene-state in scenario 6). These two patients groups were therefore combined in our approach.

#### *XPNPEP2*

Patients were detected with significant unfavourable clinical outcome for OS (Figure 2E, Table 1) and EFS (Additional file 2E, and 3) if the promoter of gene X-Prolyl Amino peptidase 2 was DNA hypermethylation and the expression was upregulated (i.e., patients with *XPNPEP2 *in scenario 1).

#### *PPARD*

The third independent prognostic marker that we detected is Peroxisome Proliferator-Activated Receptor Delta (*PPARD*). We detected significant favourable clinical outcome for OS (Figure 2F, Table 1) and EFS (Additional file 2F, and 3), among patients with hypomethylated levels and without significant change of gene expression levels (i.e., patients with *XPNPEP2 *in scenario 6). In addition, favourable clinical outcome was also seen for patients with promoter hypomethylation and upregulated gene expression levels (i.e., patients with the *PSMD2 *gene-state in scenario 4). These two patients groups could therefore be combined.

Taken together, by analysing methylation and expression data together we could associate three more WNT signaling genes with prognostic relevance, enforcing the role of WNT signaling in leukemia. A comparison of the integrative results with the use of only gene expression profiles revealed that only *XPNPEP2 *was significantly association with OS and EFS in univariate and multivariate analysis, i.e., upregulated expression levels (combined scenario 1, 4 and 7) are associated with unfavourable clinical outcome compared to the upregulated expression levels (combined scenario 2, 5 and 8, additional file 4C, D). On the other hand, the use of solely DNA-methylation profiles showed significant associations with clinical outcome for *PSMD2 *(additional file 4E, F), and *PPARD *(additional file 4G, H) by a comparison of hypermethylation (combined scenario 1, 2 and 3) versus hypomethylation (combined scenario 4, 5, 6). For both cases, promoter hypermethylation is associated with unfavourable outcome.

### Hierarchical clustering of AML patients based on gene-state scenarios revealed grouping of patients that are associated with clinical outcome

It has been shown previously that patients with two or more hypermethylated WNT genes are associated with unfavourable survival compared to patients with zero or one hypermethylated WNT genes [8]. We therefore asked ourselves whether we can improve the subtyping of patients based on the patterns of multiple WNT signaling genes. We hierarchically clustered the patients, using the 13 WNT signaling genes that are previously associated with leukemia, with a hamming distance over the gene-state description and complete linkage (Figure 1A). Eight clusters are detected in total from which four clusters showed enrichment with known cytogenetic or molecular abnormalities, and three patient clusters showed enrichment with DNA hypermethylation. We focussed only on cluster 1 and 5 as these showed significant differences in clinical outcome for both OS and EFS compared to the patients in any of the other clusters.

#### Cluster 1

Cluster 1 is one of the three clusters (together with cluster 2, and 8) that showed significant overrepresentation of genes with DNA hypermethylation. However, only cluster 1 resulted in significant unfavourable clinical outcome for OS and EFS (Figure 1B, C and Additional file 5). The two other clusters are enriched for the good cytogenetical risk group (cluster 2), and for MDS patients (cluster 8). DNA hypermethylation of WNT signaling genes among the good cytogenetical risk group [8], and MDS/secondary AMLs [5,19] are supported by previous results. In fact, it has also been illustrated that patients with multiple hypermethylated WNT signaling genes are associated with unfavourable clinical outcome [8]. But we demonstrate that significant unfavourable clinical outcome is only seen for patients with overrepresentation of genes in scenario 3 (DNA hypermethylation without significant change of gene expression levels). Patients in this cluster (cluster 1) also showed significant occurrence of *ASXL1 *mutations, which has previously been described to be a prognostic marker for unfavourable outcome [20]. To determine whether the DNA hypermethylation (genes in scenario 3) causes the unfavourable outcome of cluster 1 patients or whether it is caused by the *ASXL1 *mutations, we included *ASXL1 *as an additional covariate in the multivariate analysis. With a result that Cluster 1 still showed significant survival for OS (*P *< 0.025, HR: 1.58) and EFS (*P *< 0.028, HR: 1.57), and thereby indicating that patients with DNA hypermethylation of WNT genes are associated with unfavourable survival.

#### Cluster 5

Patients in cluster 5 also showed unfavourable OS and EFS (Figure 1D, E). These patients were enriched for genes with their state in scenario 8 but showed no enrichment for other cytogenetical or molecular abnormalities.

With the cluster analysis we illustrate the complex behaviour of multiple WNT signaling genes and their combined association with clinical outcome. Using both the methylation and expression profiling we are able to refine current subtyping of AML patient's giving new leads to further research.

### The good risk patients group is characterized by DNA hypermethylation of the WNT pathway

**Figure 3:**
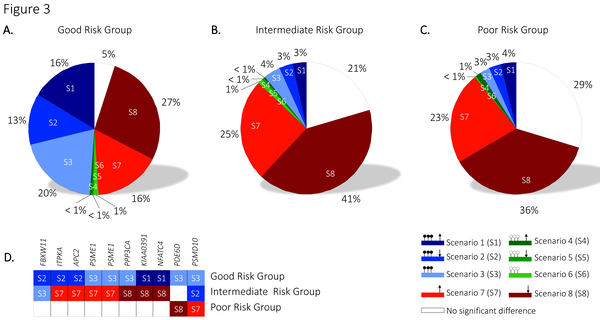
**The distribution of gene scenarios, and gene specificity among the three AML risk groups**. Classification of the 268 WNT signaling genes into the mutually exclusive scenarios for (A) good risk group, (B) intermediate risk group, and (C) poor risk group. (D) Genes with unique gene-state scenario among the AML risk groups.

We analysed the gene expression and DNA-methylation profiles of the 268 WNT signaling genes to infer whether WNT signaling genes could explain differences among the risk groups. We detected that DNA hypermethylation (genes whose state is in the scenarios 1, 2, or 3) is significantly more seen in the WNT genes among the good risk group patients when compared to the intermediate and poor risk group (Figure 3A-C, blue shaded colours). By looking into the gene-states of the WNT genes, we found that 33% of the WNT genes are in similar gene-states for all the three risk groups, whereas only 9 genes (10 refseqs) showed unique gene-states for the risk groups (Figure 3D). Among them 'proteasome 26S subunit, non-ATPase, 10' (*PSMD10*). For *PSMD10 *there is evidence that positive expression levels associate with short survival time of patients in pancreatic ductal adenocarcinoma tissues [21]. This is in line with our results where the poor risk group is detected with overexpression, and the good and intermediate risk group with promoter DNA hypermethylation of *PSMD10*.

### Good risk patients group shows interactions between WNT signaling genes and genes of the proteasome network

To gain more insights in the role of DNA-methylation for the signaling cascade of WNT we examined whether the methylation status associates with the functional importance of a gene. To get a notion of the functional importance of a gene, we assumed that the degree of a gene, in a co-expression network is indicative for its role: i.e., a gene with frequent interactions potentially regulates (or is regulated by) more genes. Consequently, we construct a co-expression network by means of pairwise Pearson correlations between the continuous mRNA expression levels for the poor, intermediate and good AML risk group (see methods).

**Figure 4:**
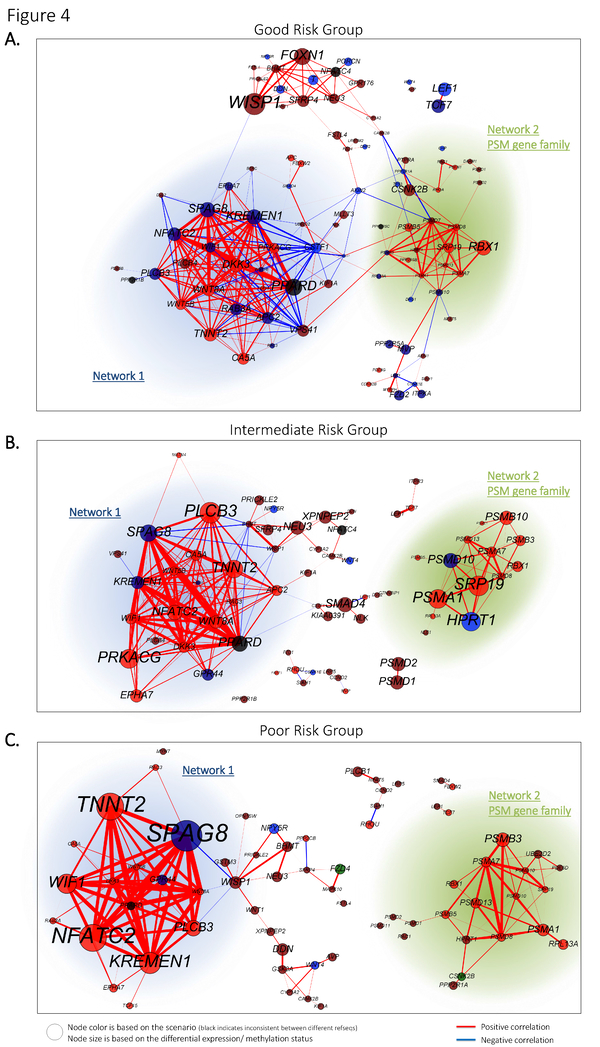
**Co-expression network among cytogenetical risk groups**. Significant expressed genes (comparison with CD34+ normal samples) among each risk group are pairwise correlated using the continuous gene expression levels. A network is subsequently build by means of the significant pairwise correlations. The colour of the nodes (genes) illustrates the scenarios, whereas the size of the node and the label text is based on the -log10(*P*-value) for differential expression or methylation. Edges with positive correlation are indicated in red, whereas negative correlations are indicated in blue. The thickness of the edges are based on the absolute correlation measure which varies between 0.6 and 1. The proteasome gene family network (PSM-family) describes members of the proteasome complex.

The good risk group contained in total 167 significantly expressed genes for which 65 are upregulated and 102 downregulated. Pairwise correlation resulted in 96 unique genes with one or more significant pairwise interactions (Figure 4A). DNA hypermethylation was detected in 36% of the genes, whereas the genes with highest degree consists of DNA hypermethylated genes, such as *KREMEN1, APC2, CSTF1, NFATC2, SPAG8, WIF1 *(Figure 4A).

The intermediate risk group contains 160 significantly regulated genes (63 upregulated, and 97 downregulated), for which 67 unique genes had significant pairwise interactions. Nine genes are detected with DNA hypermethylation from which *KREMEN1, and SPAG8 *are listed with the highest degree (Figure 4B). The poor risk group contains 56 significantly upregulated and 89 downregulated genes for which 68 unique genes had significant pairwise interactions. The genes with the highest degree contained, among others, the DNA hypermethylated gene *SPAG8 *(Figure 4C).

Interestingly, genes with a high degree appear to be DNA hypermethylation, such as for gene *KREMEN1*, which is seen in similar state in the good and intermediate risk group but not in the poor risk group. *KREMEN1 *encodes a high-affinity dickkopf homolog 1 (*DKK1*) transmembrane receptor that functionally cooperates with *DKK1 *to block wingless (WNT)/beta-catenin signaling. Another finding is that each risk group contains two similar sub-networks (i.e., network 1 and 2, Figure 4), indicating that genes behave, to some extent similar. The first network consists of genes such as *KREMEN1*, *SPAG8*, *DKK3*, and *NFATC2 *that appeared to be under control of DNA hypermethylation in the good risk group, but not for the intermediate and poor risk group. The second network is significantly enriched for proteasome gene family members (PSM) which provides instructions for making parts of the proteasomes, and are part of the cell's quality control system. Remarkably, the good risk group has frequent co-regulatory effects with the proteasome network whereas this seems absent in the intermediate and poor risk group. To summarize, we show that DNA hypermethylated genes appear to be highly connected in the co-expression network, and interact with a proteasome network for the good risk group, which is not the case for the intermediate and poor risk group.

## Conclusions

We created a computational approach to classify genes into scenarios that describe, in a discrete manner, the state of a gene considering its gene expression and the DNA-methylation levels of its promoter region. Throughout the whole study we used these scenarios to analyse WNT signaling genes for patients with Acute Myeloid Leukemia. As in previous studies [6], we also detected high frequency of DNA hypermethylation of WNT genes in AML but could now better characterize patients by jointly analysing the DNA-methylation and gene expression profiles.

We illustrate that the integration of DNA-methylation and gene expression into scenarios can refine and detect novel prognostic gene markers. This is especially important when groups are selected using prognostic markers for targeted drug therapy. Using our integrative approach we detected in total seven independent prognostic markers from which two (*SFRP2 *and *XPNPEP2*) overlapped if only gene expression profiles are used (out of nine). Based on only DNA-methylation profiles, 14 prognostic markers are detected from which two (*PSMD2 *and *PPARD*) overlapped with our integrative approach (Additional file 6).

Our approach also refined patient groups for which previously was shown that multiple hypermethylated WNT genes were associated with unfavourable survival [8]. We demonstrate that unfavourable survival of patients is associated with promoter hypermethylation without change of gene expression levels (scenario 3). In addition, we detected that good risk group patients significantly harbour DNA hypermethylation (which is in line with previously results [12], but we provide a mechanistic understanding of the functional role of DNA hypermethylation by examining the co-expression gene networks. Although the exact functional relationships of DNA-methylation and the proteasome is difficult to comprehend, the importance of proteasome inhibitors is described for Multiple Myeloma, where the use of proteasome inhibitors are potentially interesting as they can serve as therapeutic strategy by directly targeting both the tumor and bone disease [22]. Their role in AML is, however, less known. Yet, proteasome inhibition is recently introduced as a promising novel anticancer therapy for AML [23]. Further, the proteasome inhibitor Bortezomib sensitizes AML with myelomonocytic/monocytic phenotype (M4/M5 AML based on FAB classification) in AML cell lines [24].

Although many studies illustrate that gene expression or DNA-methylation levels in isolation can serve as a prognostic marker, our computational approach proved effective in the identification of prognostic markers and improves characterization of patient groups by the integration of gene expression and DNA-methylation profiles. This is especially important when groups are selected on prognostic markers for targeted drug therapy.

## Methods

### Patient samples

Diagnostic bone marrow (BM) or peripheral blood (PB) samples from 344 adults were analysed. All patients provided written informed consent in accordance with the Declaration of Helsinki. All trials were approved by the Institutional Review Board of Erasmus University Medical Center. Summary of clinical, cytogenetical and molecular features of the patient are previously described [9].

### Genome-wide high throughput data

Two high throughput datasets were used in this study: genome-wide mRNA expression profiling data (GEP) and for the same 344 samples, genome-wide DNA-methylation profiling data (DMP). In addition, eight GEP and eleven DMP reference samples (CD34+) are available. These two assays provide mRNA expression and promoter DNA-methylation abundance levels. GEP data is generated using Affymetrix HGU133 plus2.0 (Santa Clara, CA, USA). Normalization of raw GEP data was processed with RMA [25,26]. In addition, probes are remapped to refseq transcripts using a custom CDF definition. Gene expression levels are mean centered over all samples. DMP-data is generated using the HELP assay and pre-processed as described previously [27]. Both data sets are annotated using UCSC hg19, and are available at the NCBI Gene Expression Omnibus [accession numbers GSE14468 and GSE18700]. Refseq transcripts from GEP and probesets from DMP are included on the following terms: 1) Probeset are annotated with chromosome information, genomic location and strand position; 2) Mapping of probesets was without known sequence variation; 3) Annotated genes for the refseq transcripts and probesets overlapped between both data sets. This resulted into 12925 uniquely overlapping genes for 14292 refseq transcripts (GEP) and 19899 probesets (DMP). However, in this study we were specifically interested in the genes that are annotated as being part of the WNT signaling pathway.

### WNT pathway signaling genes are derived from the Molecular Signature database

Eight WNT associated pathways are utilized from the Molecular Signature Database (MSigDB, v3.0), and we derived 364 unique genes from them: Biocarta WNT Pathway (26 genes), KEGG WNT Signaling Pathway (151 genes), Morf WNT1 (101 genes), Reactome Signaling by WNT (58 genes), ST WNT Beta Catenin Pathway (31 genes), ST WNT CA2 Cyclic GMP Pathway (19 genes), Willert WNT Signaling (20 genes), WNT Signaling (89 genes). From the 364 WNT associated genes, 268 were available in the gene expression profiling data (291 refseqs), and overlapped with the DNA-methylation profiling data (539 probesets).

### Discretization of DNA-methylation and mRNA expression profiles into gene-state scenarios for individual cancer samples

Discretization of the data was used to categorize each gene (refseq) into one of nine mutually exclusive scenarios. A scenario describes in a discrete manner the change in gene expression (upregulation, downregulation or no regulation) and of the same gene, the change in DNA-methylation level (hypermethylation, hypomethylation or no methylation). Classification of genes in one of the scenarios was accomplished by first discretising the continuous gene expression profiles in comparison to CD34+ control samples (Additional file 1A,B). A gene transcript (refseq) is marked as upregulated/downregulated if the mRNA expression levels were lower/higher than the 99% confidence interval of the CD34+ normal bone marrow samples of the same gene transcript. Otherwise, a gene transcript is marked as no difference in mRNA expression. Similarly, the continuous DNA-methylation profiles were also discretized in comparison to CD34+ normal bone marrow samples (Additional file 1A,B). A probeset is marked as hypomethylated/hypermethylated if the DNA-methylation levels were higher/lower than the 99% confidence interval of the CD34+ normal bone marrow samples of the same probeset. Otherwise, a gene is marked as no DNA-methylation. Both discretized data sets are subsequently integrated to define the gene status in terms of scenarios (Additional file 1C). There are nine possible scenarios in total. The first three scenarios [1-3] describe DNA hypermethylation with mRNA transcript expression (up, down or no/low expression), and are colored in different shades of blue. Scenarios [4-6], describe DNA hypomethylation with mRNA transcript expression (up, down or no/low expression), and are colored in different shades of green. Scenarios [7,8] describe upregulation and downregulation but without difference in DNA-methylation levels, and are colored in different shades of red. The ninth scenario contains genes for which no difference in gene expression and no difference in DNA-methylation levels were observed, or cases for which the gene promoters contained multiple probesets with inconsistent DNA-methylation levels (e.g., one probeset with hypermethylated whereas another with hypomethylated, independent of the gene expression level). We will denote this scenario as no difference, and is colored as white in the figures. The colors are consistently used throughout the whole manuscript.

### Discretization of DNA-methylation and mRNA expression profiles into gene-state scenarios for patient risk groups

Discretization and integration of gene expression and DNA-methylation data of the cytogenetical risk groups (poor, intermediate and good) into gene-state scenarios is performed in two steps. The first step involves comparison of the GEP patient data with GEP reference samples (CD34+), and the DMP patient data with DMP reference (CD34+) data by means of a student T-test. Significant genes (FDR<0.01) with a significant positive T-statistic are categorized as upregulated/hypomethylated and with a significant negative T-statistic as downregulated/hypermethylated. For DNA-methylation probesets, the absolute fold difference must also be ≥1.5 compared to the reference samples in order to have a sufficient effect-size. The second step is the integration of the GEP and DMP gene status in terms of scenarios. This is similar as described for the individual cancer samples, thus each gene is classified into one of the nine scenarios based on the DNA-methylation with mRNA transcript expression level changes.

### Construction of the co-expression networks

To construct the co-expression network and determine the gene-degree, we applied the following approach: 1) gene expression levels among the AML risk groups must be significantly upregulated or downregulated compared to the CD34+ normal samples (as described in the method section); 2) pairwise Pearson correlations among the continuous mRNA gene expression levels are computed between the significantly detected WNT signaling genes; 3) gene interactions with r>0.6 and *P *< 0.05 are marked, and used in the network; 4) the degree for each gene is determined by the number of edges it contains; 5) each gene in the network is illustrated in terms of the mutually exclusive scenarios.

### Survival analysis based on gene-state scenarios

To assess whether a gene is significantly associated with clinical outcome, we compared patient groups based on their gene-state scenarios (Additional file 1D). To prevent high multiple test correction by comparing a maximum of nine patient groups (i.e., patients that are categorized in each of the nine scenarios), we first clustered patient groups on survival. We applied the following approach: 1) for each gene a pairwise comparison between patient groups (e.g., among the gene-state scenarios) is performed using the log-rank test (for OS or EFS) (Additional file 1E). The pairwise log-rank test is then used as a distance matrix (indicating the similarity between patient groups survival) which is in turn hierarchically clustered using Euclidean distance and average linkage. The dendrogram is then cut horizontally through the leaves at the smallest height that results in two clusters (Additional file 1F). The resulting two clusters, each containing one or multiple patient groups are subsequently compared against each other for its difference in OS and EFS using univariate (log-rank test) and multivariate analyses (Cox's proportional hazard ratio model) (Additional file 1G). For the multivariate analysis we adjusted for the covariates; age, white blood cell count, *CEBPA^double−mutation^*, *NPM1^mutation^*, and *FLT3^ITD ^*status. Note that the definition of survival endpoints were based on the recommended consensus criteria [28].

## Competing interests

The authors declare that they have no competing interests.

## Authors' contributions

ET implemented the methodologies, analysed, interpreted the data, and drafted the manuscript. ET, FJTS, and MJTR participated in the design of the study and contributed to the writing of the paper. All authors provided relevant input at different stages of the project, and read and approved the final manuscript.

## Supplementary Material

Additional file 1 **Schematic overview: Discretization of DNA-methylation and mRNA expression profiles into gene-state scenarios and survival analysis**. (A) Discretization of the continuous gene expression and DNA-methylation profiles compared to CD34+ control samples. (B) A gene transcript (refseq) is marked as upregulated/downregulated if the mRNA expression levels were lower/higher than the 99% confidence interval of the CD34+ normal bone marrow samples of the same gene transcript. Similarly, the continuous DNA-methylation profiles were also discretized in comparison to CD34+ normal bone marrow samples. (C) Both discretized data sets are subsequently integrated to define the gene status (scenarios) in terms of mRNA transcript expression and DNA hypermethylation. (D) Grouping of samples that have the same gene-state scenario. In this example, Gene 1 groups patients in scenario S1, S2, S4, S7 and S8. (E) Pairwise comparison between patient groups using the log-rank test (for OS or EFS). (F) The distance matrix based on the pairwise log-rank test is hierarchically clustered, and cut horizontally through the leaves that results in two clusters. (G) The resulting two clusters, each containing one or multiple patient groups are subsequently compared against each other for its difference in OS and EFS using univariate (log-rank test) and multivariate analyses (Cox's proportional hazard ratio model). Click here for file

Additional file 2 **Kaplan-Meier curves for the identified prognostic genes for Event-Free Survival**. The Kaplan-Meier curves illustrates the prognostic markers and the patient groups based on the classification of gene scenario. (A) *LEF1*, (B) *SFRP2*, (C) *RUNX1*, (D) *PSMD2*, (E) *XPNPEP2*, (F) *PPARD*, and (G) *AXIN2*. Significance for each prognostic marker is computed by comparing patients in the particular scenario versus patients in any other scenario (black line) using a univariate (depicted by the *P*-value), and multivariate analysis (depicted by the adjusted *P*-value). Click here for file

Additional file 3 **Multivariate analysis for the identified prognostic genes for Event-Free Survival (EFS)**. Cox proportional hazard model for multivariable analyses of prognostic markers for Event-free survival. Analyses included 344 AML patients. Abbreviations: HR, hazard ratio; CI, confidence interval; *α CEBPA^double−mutation ^*status versus *CEBPA^wt^*, *β FLT3^ITD ^*versus no *FLT3^ITD ^*mutation, *β NPM1^mutant ^*versus *NPM1^wt^*, *δ *WBC count higher than 20 × 10^9^/*L *versus lower than 20 × 10^9^/*L*, $ Age is used as continuous variable. Click here for file

Additional file 4 **Kaplan-Meier curves by using solely gene expression or DNA-methylation profiles**. The Kaplan-Meier curves illustrates the prognostic markers and the patient groups based on the comparison upregulated (scenario 1,4,7) versus downregulated (scenario 2,5,8) gene expression levels, and hypermethylated (scenario 1,2,3) versus hypomethylated (scenario 4,5,6) levels. Significance is assessed by comparing patients using a univariate (depicted by the *P*-value), and multivariate analysis (depicted by the adjusted *P*-value). Click here for file

Additional file 5 **Multivariate analysis for cluster 1 and cluster 5 for OS and EFS**. Cox proportional hazard model for multivariable analyses of cluster 1 and cluster 5 for OS and EFS. Abbreviations: HR, hazard ratio; CI, confidence interval; *α CEBPA^double−mutation ^*status versus *CEBPA^wt^*, *β FLT3^ITD ^*versus no *FLT3^ITD ^*mutation, *β NPM1^mutant ^*versus *NPM1^wt ^*, *δ *WBC count higher than 20 × 10^9^/*L *versus lower than 20 × 10^9^/*L*, $ Age is used as continuous variable. Click here for file

Additional file 6 **Venn diagram illustrating the overlap of prognostic markers detected by the integrative approach, the use of solely gene expression profiles, and solely DNA-methylation profiles**. Using the integrative approach, seven prognostic markers are detected. Using solely gene expression profiles, 9 prognostic markers are detected. Using solely DNA-methylation profiles, 14 prognostic markers are detected. Click here for file

## Declarations

This work is part of the BioRange program of the Netherlands Bioinformatics Centre (NBIC), which is supported by the Netherlands Genomics Initiative (NGI).

This article has been published as part of *BMC Bioinformatics *Volume 16 Supplement 4, 2015: Selected articles from the 9th IAPR conference on Pattern Recognition in Bioinformatics. The full contents of the supplement are available online at http://www.biomedcentral.com/bmcbioinformatics/supplements/16/S4.
