# Supplementary figures and images for: An integrated approach of gene expression and DNA-methylation profiles of WNT signaling genes uncovers novel prognostic markers in Acute Myeloid Leukemia
Source: BMC Bioinformatics. 2015 Feb 23;16(Suppl 4):S4. doi: 10.1186/1471-2105-16-S4-S4 (PMC4347618; doi:10.1186/1471-2105-16-S4-S4)

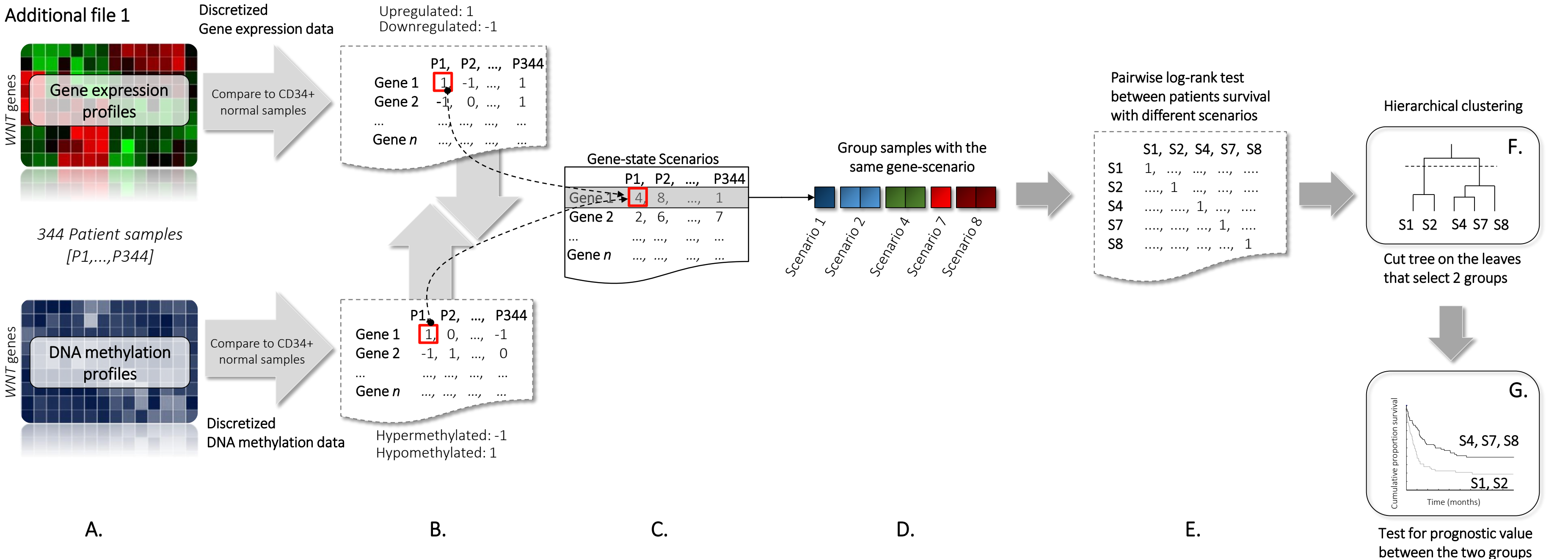

Supplement: Additional file 1 — Schematic overview: Discretization of DNA-methylation and mRNA expression profiles into gene-state scenarios and survival analysis. (A) Discretization of the continuous gene expression and DNA-methylation profiles compared to CD34+ control samples. (B) A gene transcript (refseq) is marked as upregulated/downregulated if the mRNA expression levels were lower/higher than the 99% confidence interval of the CD34+ normal bone marrow samples of the same gene transcript. Similarly, the continuous DNA-methylation profiles were also discretized in comparison to CD34+ normal bone marrow samples. (C) Both discretized data sets are subsequently integrated to define the gene status (scenarios) in terms of mRNA transcript expression and DNA hypermethylation. (D) Grouping of samples that have the same gene-state scenario. In this example, Gene 1 groups patients in scenario S1, S2, S4, S7 and S8. (E) Pairwise comparison between patient groups using the log-rank test (for OS or EFS). (F) The distance matrix based on the pairwise log-rank test is hierarchically clustered, and cut horizontally through the leaves that results in two clusters. (G) The resulting two clusters, each containing one or multiple patient groups are subsequently compared against each other for its difference in OS and EFS using univariate (log-rank test) and multivariate analyses (Cox's proportional hazard ratio model). [file 1471-2105-16-S4-S4-S1.pdf]

# Additional file 2

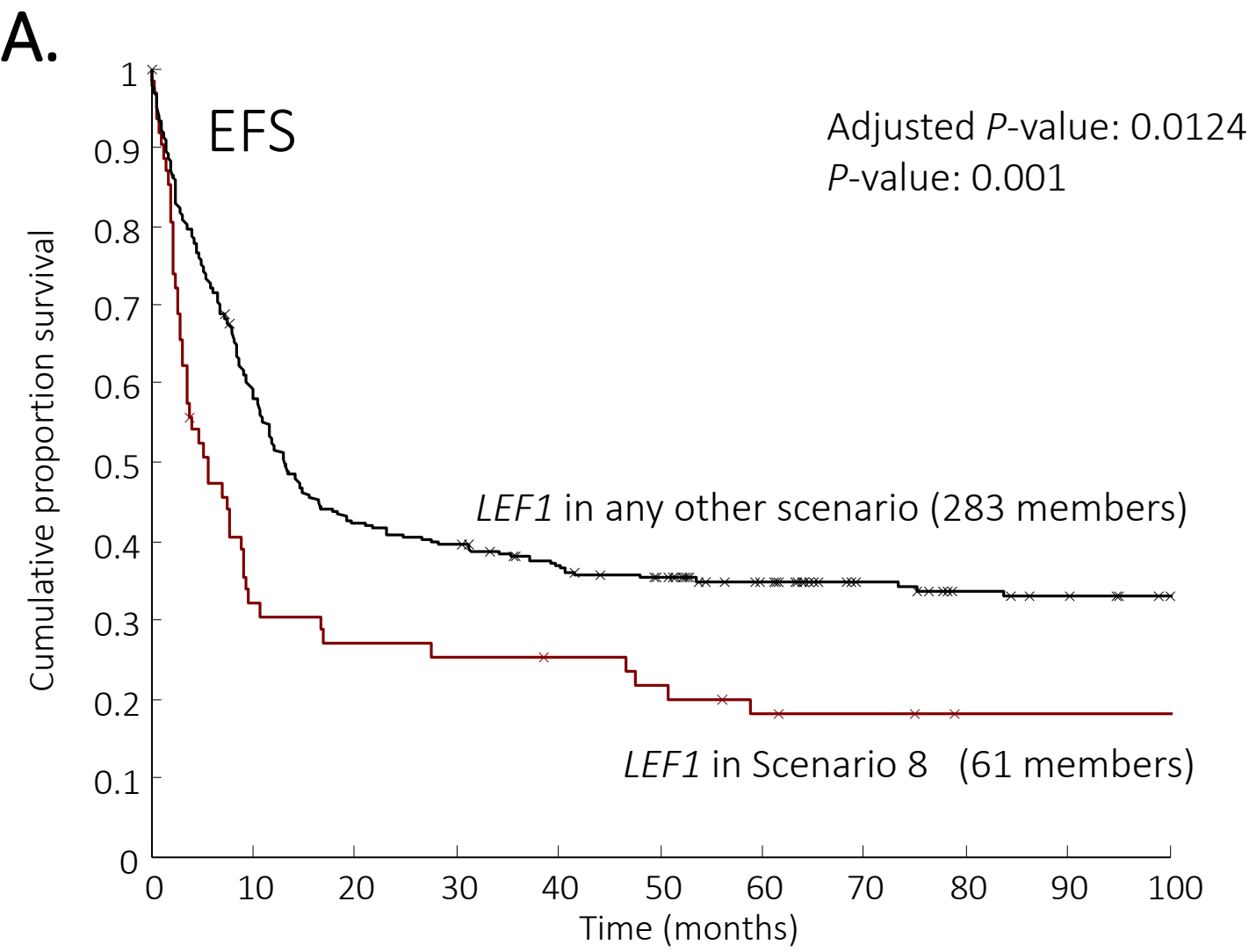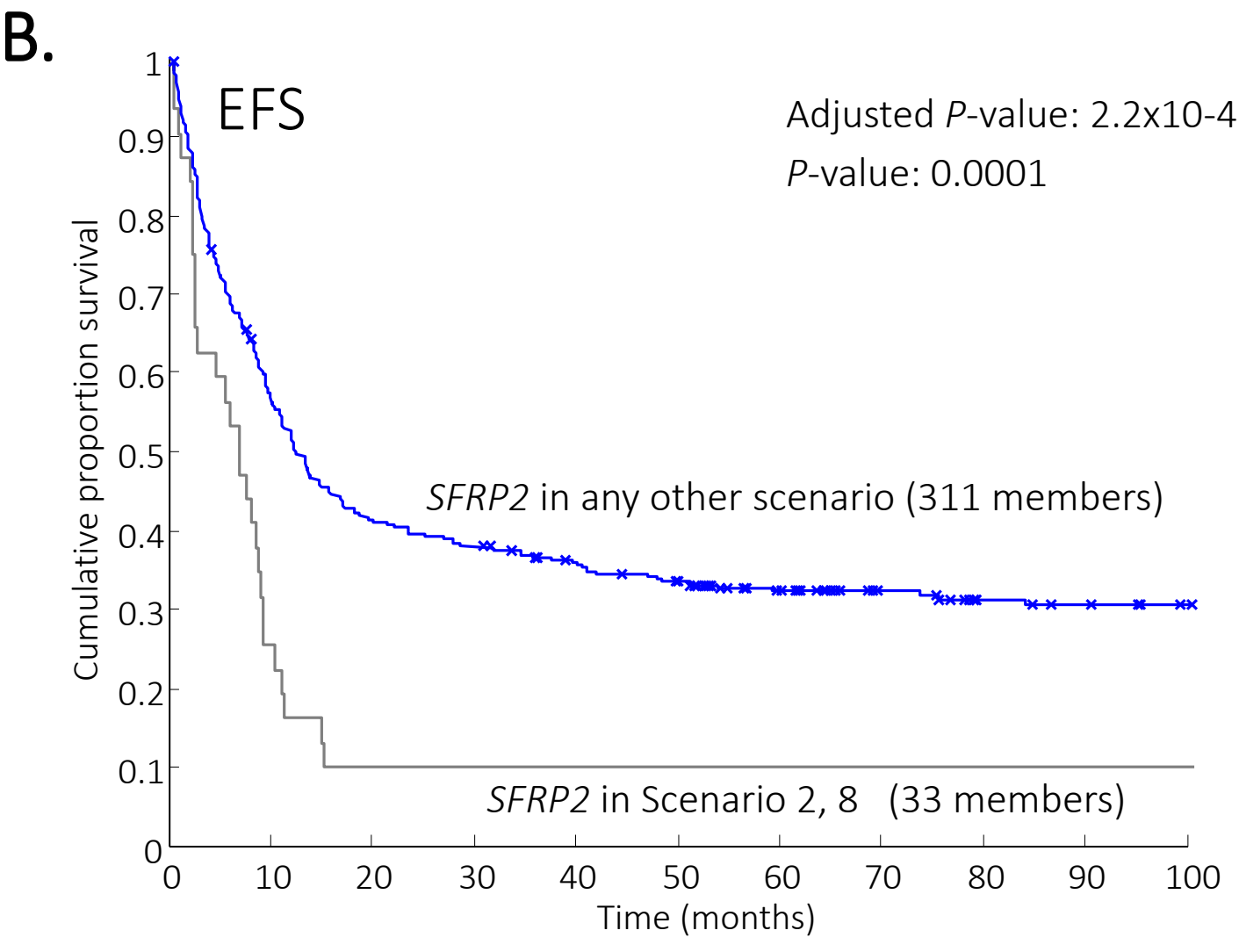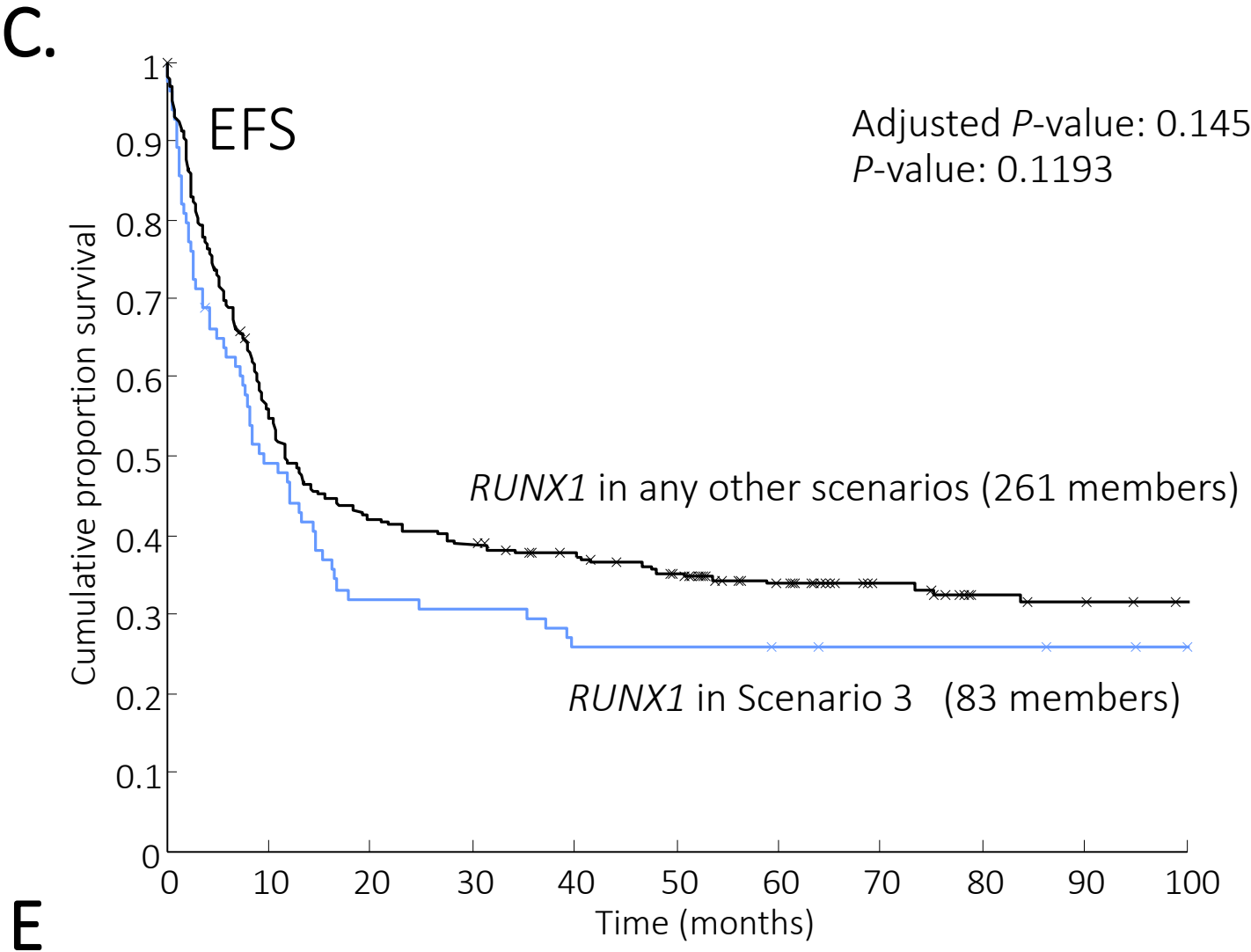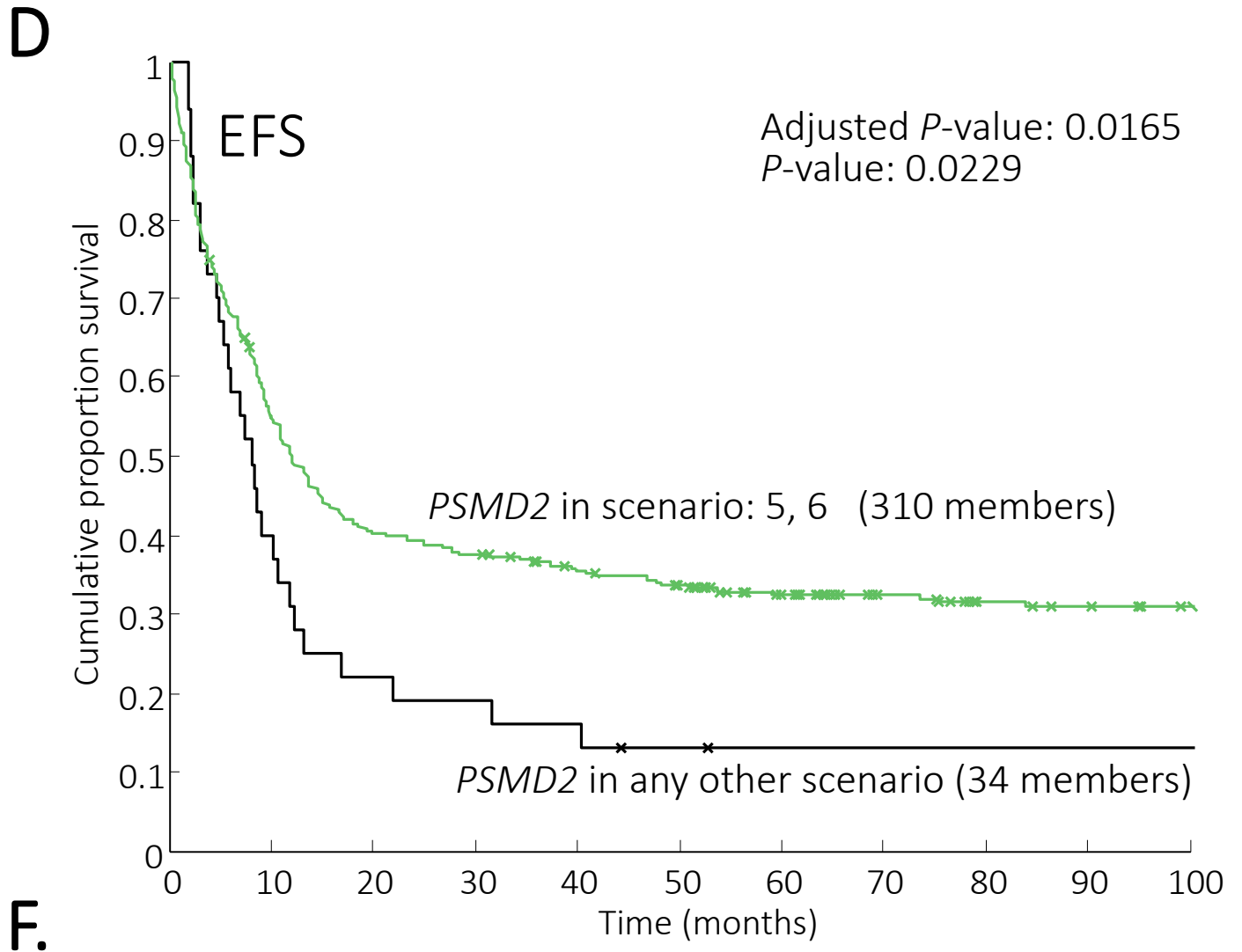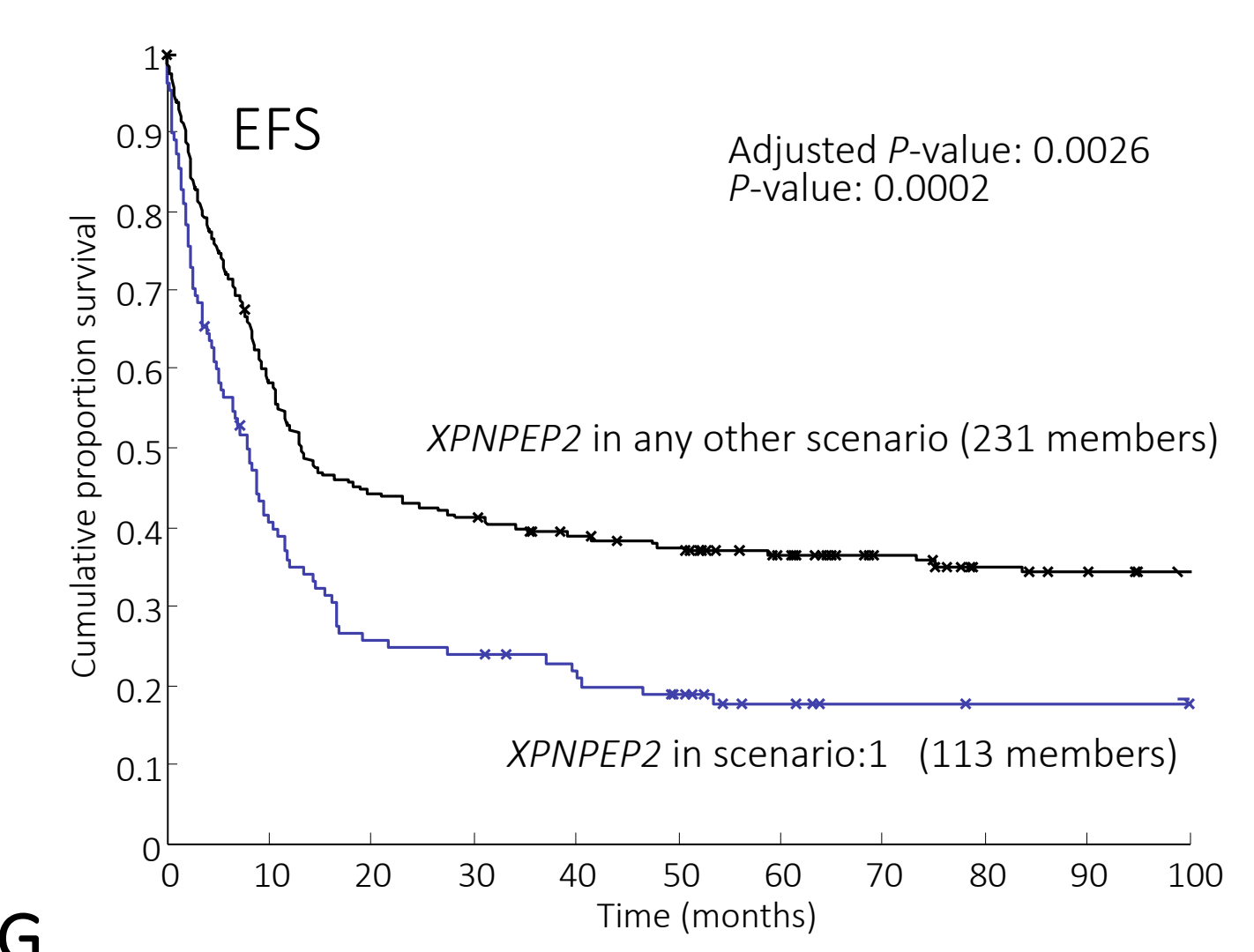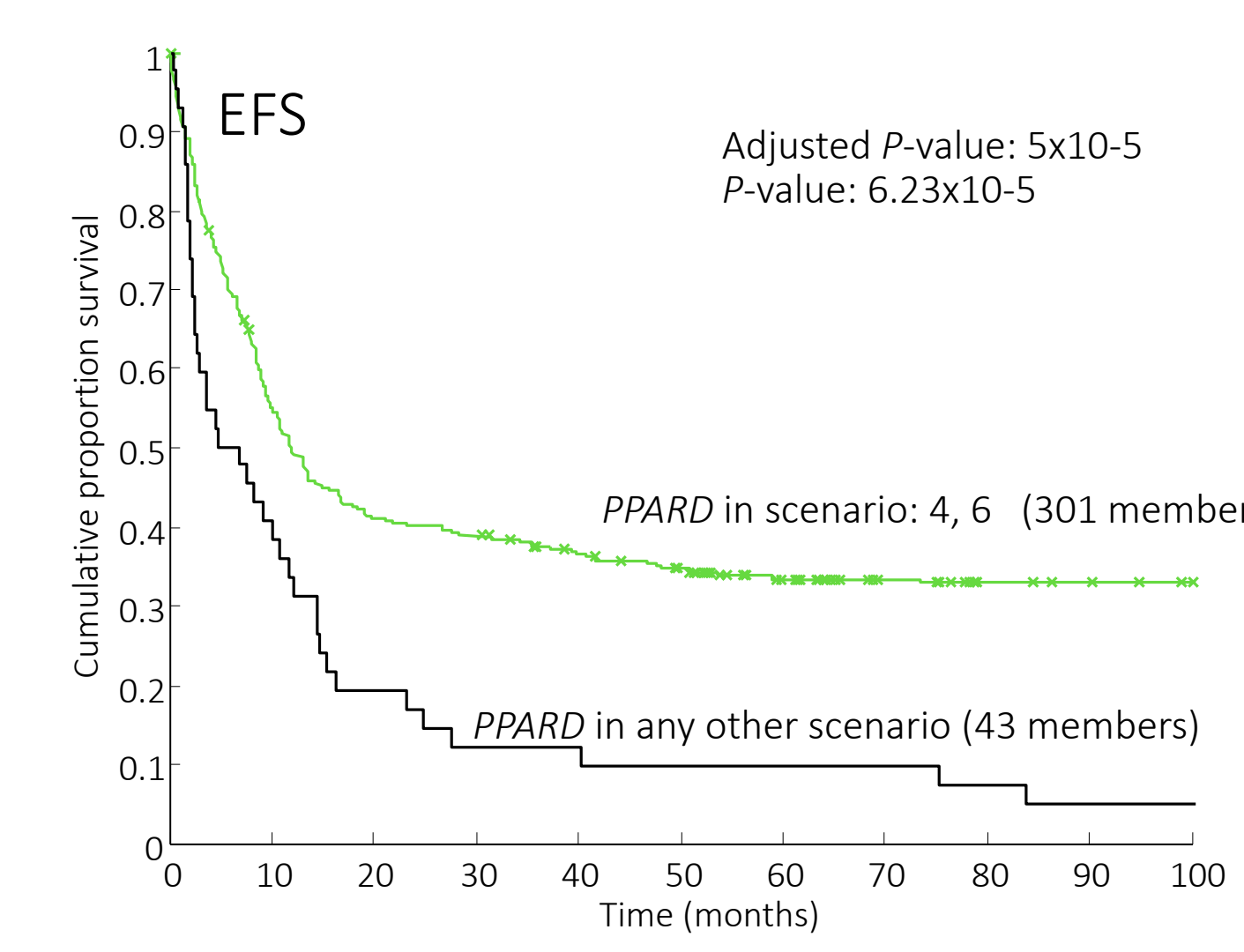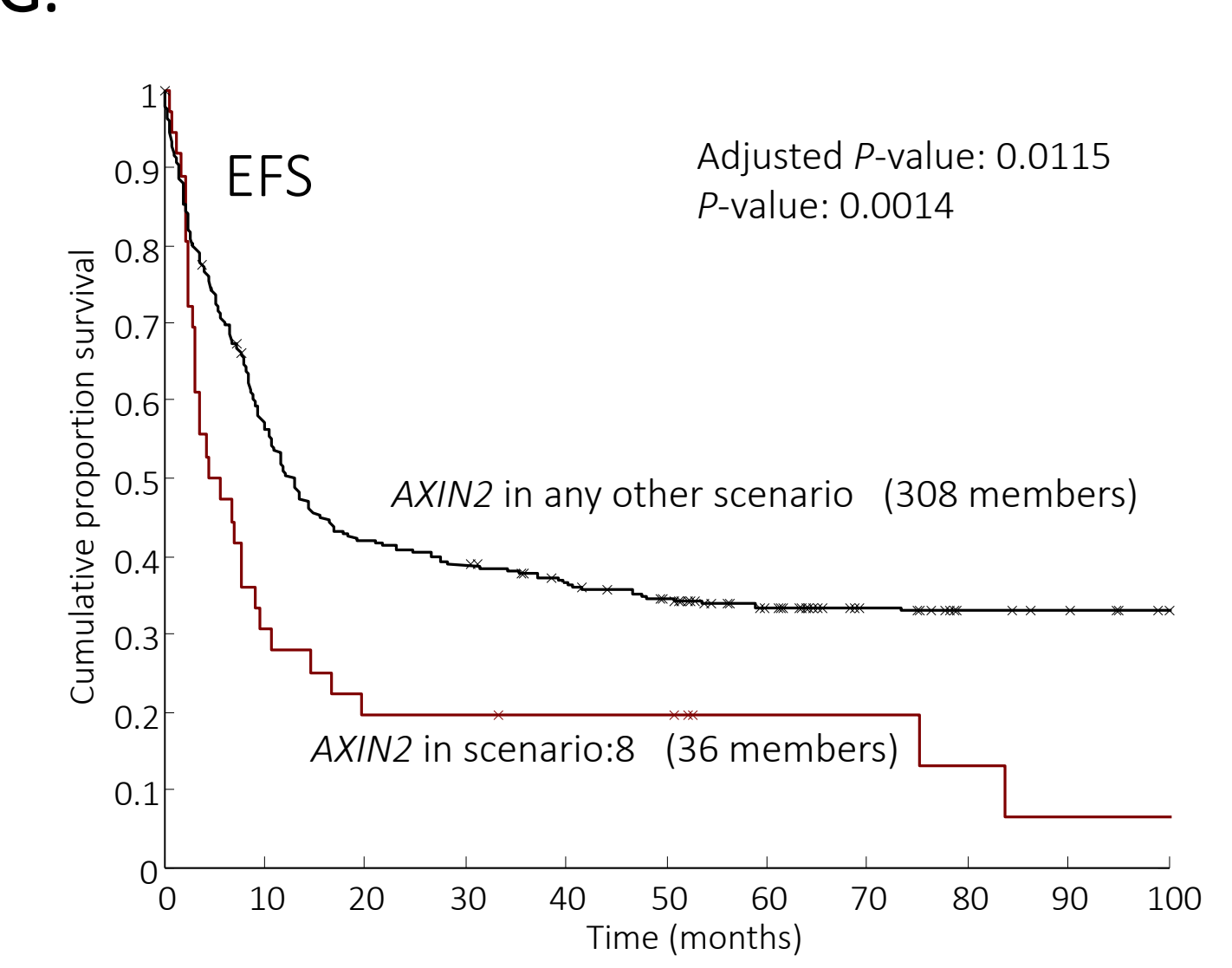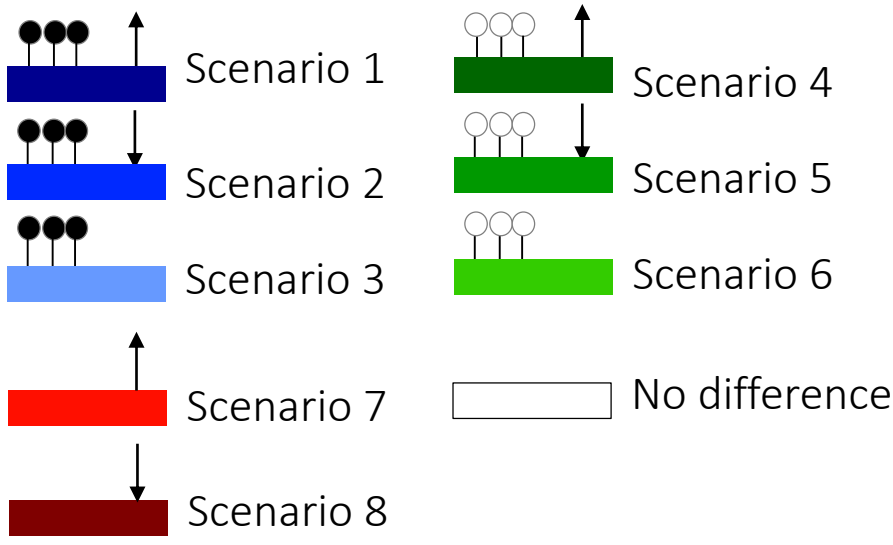

Supplement: Additional file 2 — Kaplan-Meier curves for the identified prognostic genes for Event-Free Survival. The Kaplan-Meier curves illustrates the prognostic markers and the patient groups based on the classification of gene scenario. (A) LEF1, (B) SFRP2, (C) RUNX1, (D) PSMD2, (E) XPNPEP2, (F) PPARD, and (G) AXIN2. Significance for each prognostic marker is computed by comparing patients in the particular scenario versus patients in any other scenario (black line) using a univariate (depicted by the P-value), and multivariate analysis (depicted by the adjusted P-value). [file 1471-2105-16-S4-S4-S2.pdf]

Additional file 4

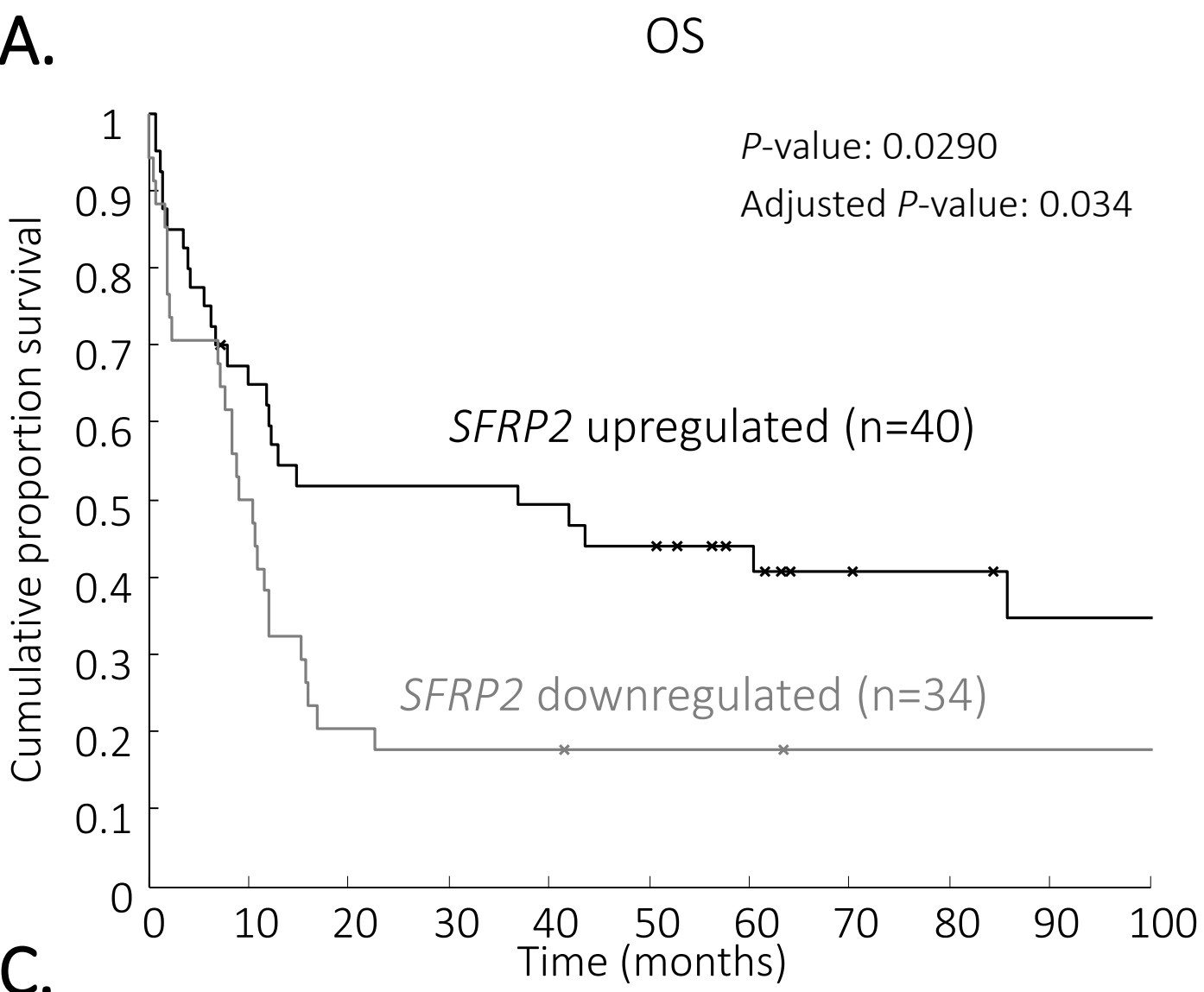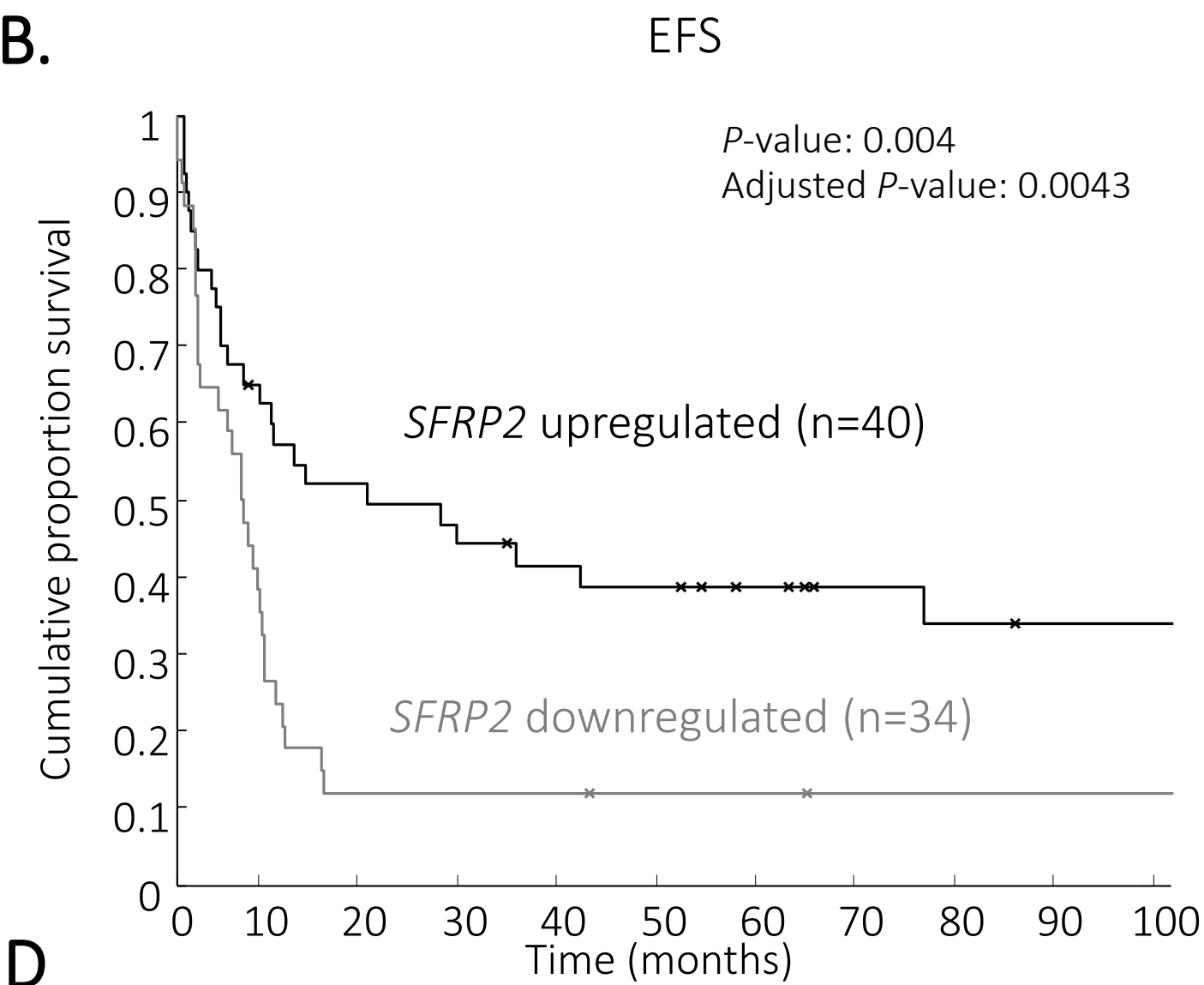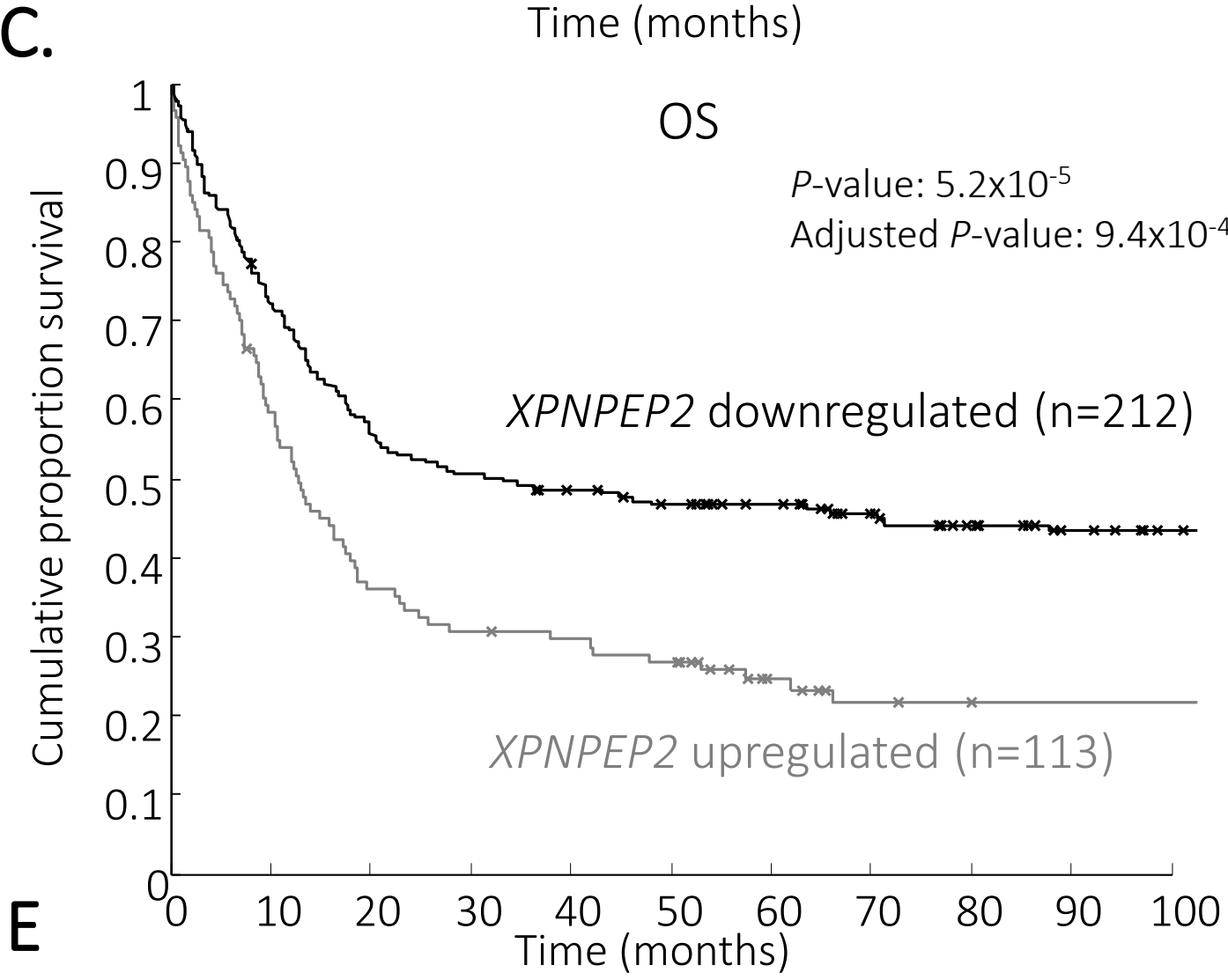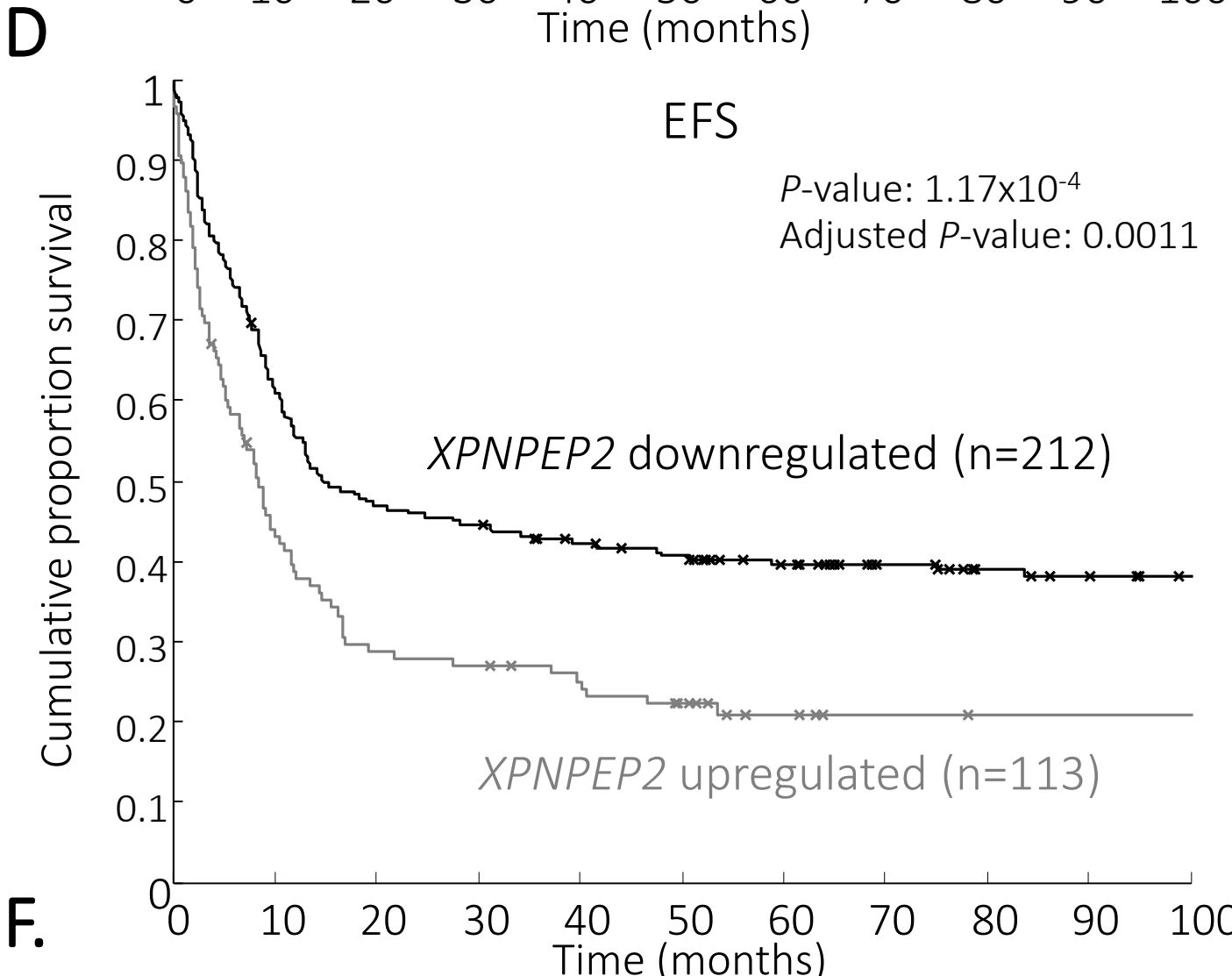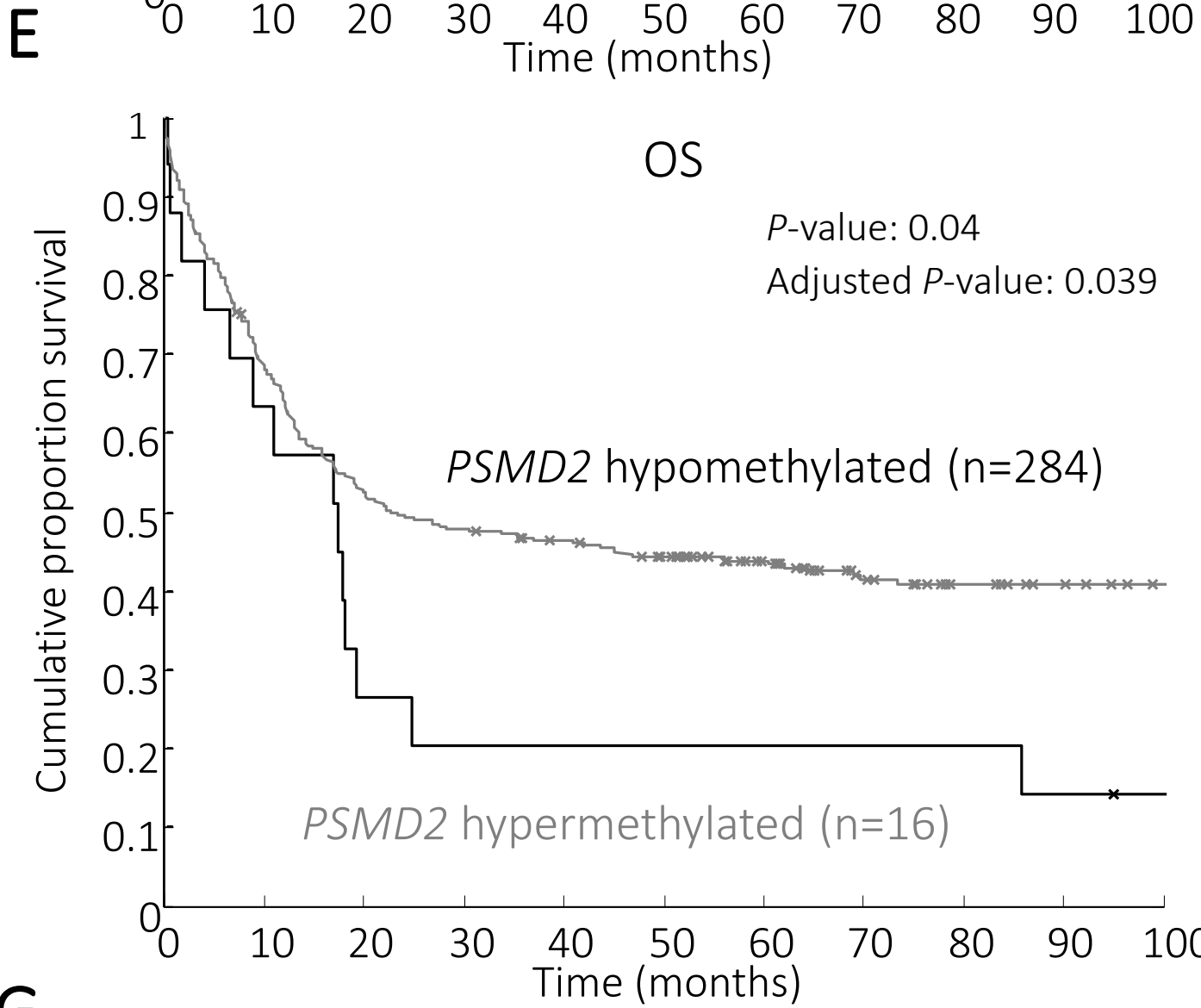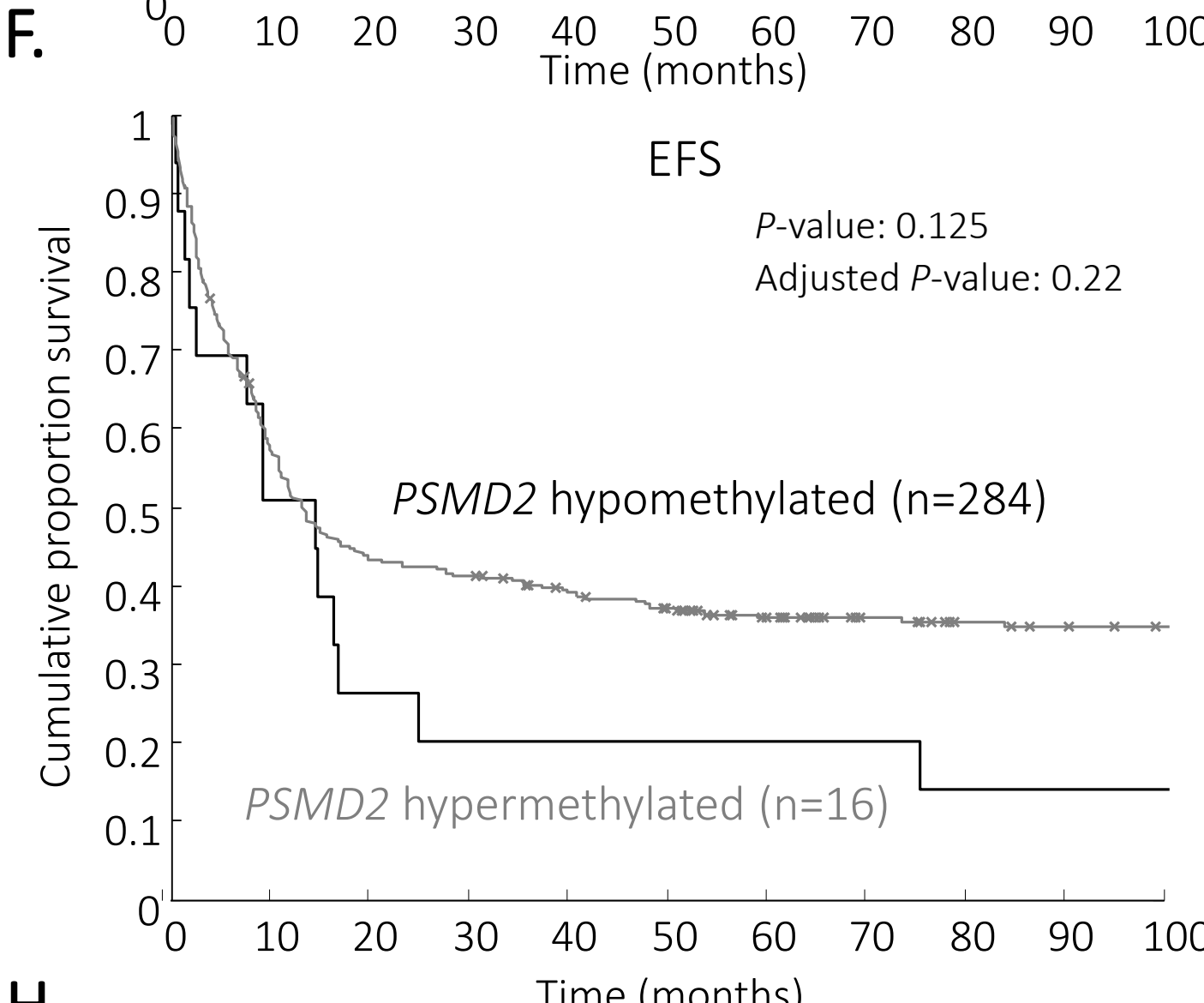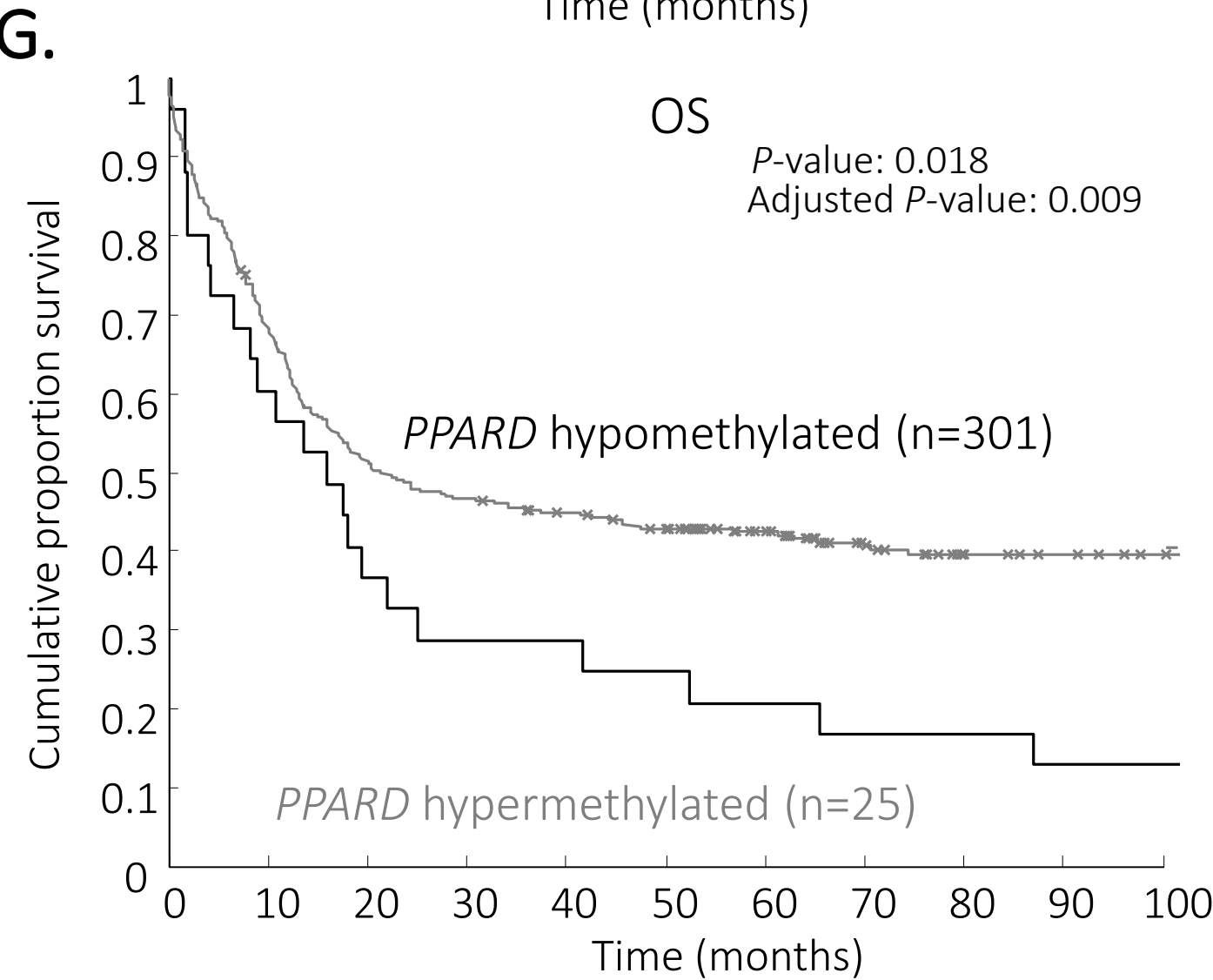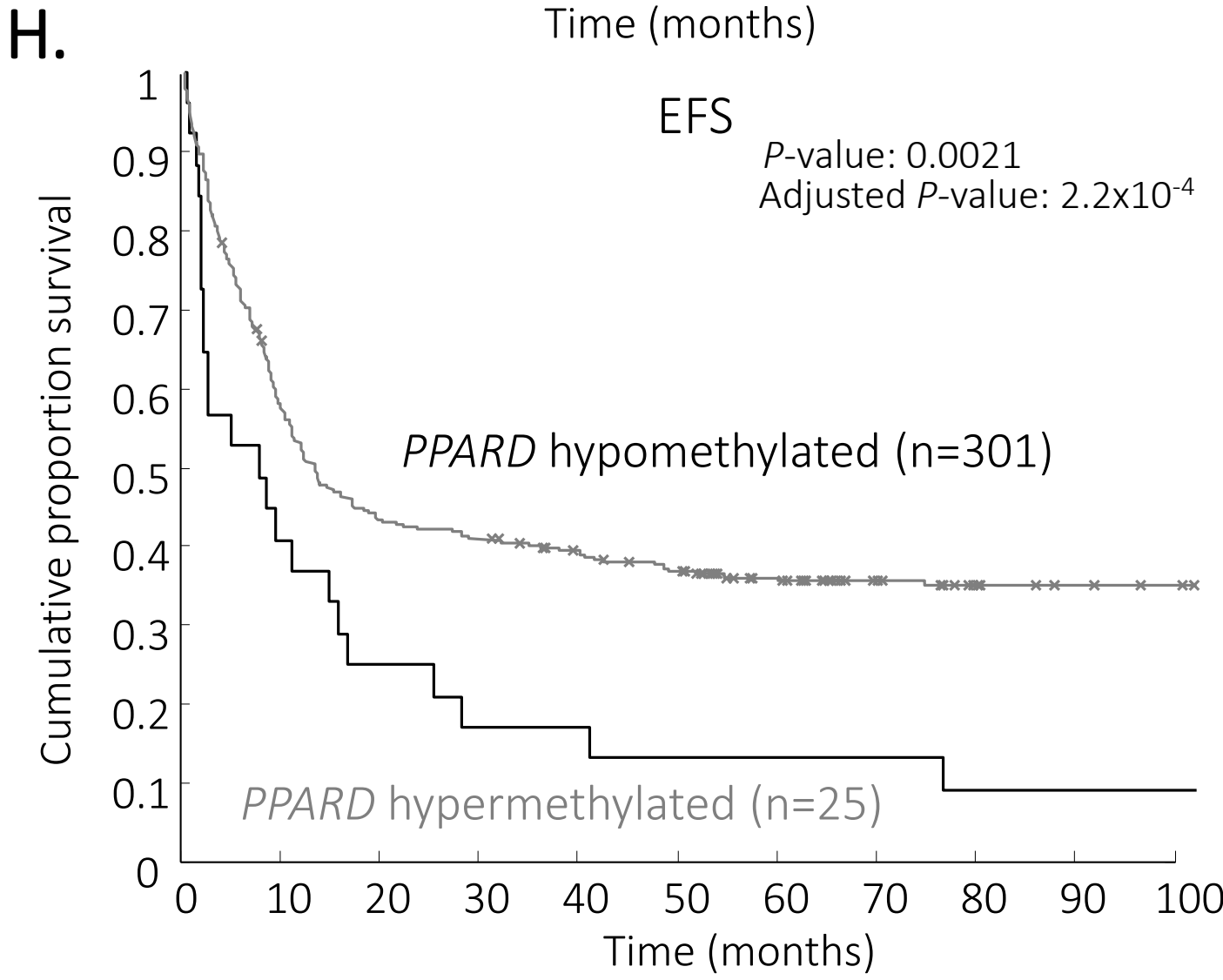

Supplement: Additional file 4 — Kaplan-Meier curves by using solely gene expression or DNA-methylation profiles. The Kaplan-Meier curves illustrates the prognostic markers and the patient groups based on the comparison upregulated (scenario 1,4,7) versus downregulated (scenario 2,5,8) gene expression levels, and hypermethylated (scenario 1,2,3) versus hypomethylated (scenario 4,5,6) levels. Significance is assessed by comparing patients using a univariate (depicted by the P-value), and multivariate analysis (depicted by the adjusted P-value). [file 1471-2105-16-S4-S4-S4.pdf]
