# Supplementary material for: An integrated approach of gene expression and DNA-methylation profiles of WNT signaling genes uncovers novel prognostic markers in Acute Myeloid Leukemia
Source: BMC Bioinformatics. 2015 Feb 23;16(Suppl 4):S4. doi: 10.1186/1471-2105-16-S4-S4 (PMC4347618; doi:10.1186/1471-2105-16-S4-S4)
Supplement: Additional file 3 — Multivariate analysis for the identified prognostic genes for Event-Free Survival (EFS). Cox proportional hazard model for multivariable analyses of prognostic markers for Event-free survival. Analyses included 344 AML patients. Abbreviations: HR, hazard ratio; CI, confidence interval; α CEBPAdouble−mutation status versus CEBPAwt, β FLT3ITD versus no FLT3ITD mutation, β NPM1mutant versus NPM1wt, δ WBC count higher than 20 × 109/L versus lower than 20 × 109/L, $ Age is used as continuous variable. [file 1471-2105-16-S4-S4-S3.pdf]

Additional file 3.

Variables	*P* -value	**HR**	95% CI-low	CI-high
Event-free survival				
LEF1 (scenario 8)	0.01247	1.51	1.09	2.08
CEBPA $^{dm\alpha}$	0.00647	0.44	0.24	0.79
FLT3 $^{ITD\ \beta}$	0.00056	1.71	1.26	2.31
NPM1 $^{+\ \beta}$	0.00003	0.51	0.37	0.70
WBC count$^{\delta}$, x10^9/L	0.00972	1.00	1.00	1.00
Age$^{\$}$	0.00501	1.01	1.00	1.02
Event-free survival				
SFRP2 (scenario 2, 8)	0.00022	2.11	1.42	3.13
CEBPA $^{dm\alpha}$	0.00550	0.43	0.24	0.78
FLT3 $^{ITD\ \beta}$	0.00012	1.83	1.35	2.49
NPM1 $^{+\ \beta}$	0.00003	0.51	0.37	0.70
WBC count$^{\delta}$, x10^9/L	0.00470	1.00	1.00	1.00
Age$^{\$}$	0.00660	1.01	1.00	1.02
Event-free survival				
RUNX1 (scenario 3)	0.14587	1.25	0.93	1.69
CEBPA $^{dm\alpha}$	0.00779	0.45	0.25	0.81
FLT3 $^{ITD\ \beta}$	0.00033	1.74	1.29	2.36
NPM1 $^{+\ \beta}$	0.00002	0.51	0.37	0.69
WBC count$^{\delta}$, x10^9/L	0.01130	1.00	1.00	1.00
Age$^{\$}$	0.00169	1.02	1.01	1.02
Event-free survival				
PSMD2 (scenario 5)	0.01651	0.71	0.54	0.94
CEBPA $^{dm\alpha}$	0.00331	0.41	0.23	0.75
FLT3 $^{ITD\ \beta}$	0.00024	1.77	1.30	2.40
NPM1 $^{+\ \beta}$	0.00004	0.52	0.38	0.71
WBC count$^{\delta}$, x10^9/L	0.00330	1.00	1.00	1.01
Age$^{\$}$	0.00426	1.01	1.00	1.02
Event-free survival				
XPNPEP (scenario 1)	0.00260	1.52	1.16	2.00
CEBPA $^{dm\alpha}$	0.00240	0.40	0.22	0.72
FLT3 $^{ITD\ \beta}$	0.00140	1.64	1.21	2.23
NPM1 $^{+\ \beta}$	0.00010	0.54	0.40	0.74
WBC count$^{\delta}$, x10^9/L	0.01530	1.00	1.00	1.00
Age$^{\$}$	0.00850	1.01	1.00	1.02
Event-free survival				
PPARD (scenario 4, 6)	0.000005	0.43	0.30	0.61
CEBPA $^{dm\alpha}$	0.00120	0.37	0.21	0.68
FLT3 $^{ITD\ \beta}$	0.00005	1.89	1.39	2.56
NPM1 $^{+\ \beta}$	0.00041	0.56	0.41	0.77
WBC count$^{\delta}$, x10^9/L	0.00670	1.00	1.00	1.00
Age$^{\$}$	0.00058	1.02	1.01	1.03
Event-free survival				
AXIN2 (scenario 8)	0.01150	1.66	1.12	2.45
CEBPA $^{dm\alpha}$	0.00270	0.40	0.22	0.73
FLT3 $^{ITD\ \beta}$	0.00045	1.72	1.27	2.33
NPM1 $^{+\ \beta}$	0.00010	0.54	0.39	0.73
WBC count$^{\delta}$, x10^9/L	0.01010	1.00	1.00	1.00
Age$^{\$}$	0.00370	1.01	1.00	1.02
