# Supplementary material for: An integrated approach of gene expression and DNA-methylation profiles of WNT signaling genes uncovers novel prognostic markers in Acute Myeloid Leukemia
Source: BMC Bioinformatics. 2015 Feb 23;16(Suppl 4):S4. doi: 10.1186/1471-2105-16-S4-S4 (PMC4347618; doi:10.1186/1471-2105-16-S4-S4)
Supplement: Additional file 5 — Multivariate analysis for cluster 1 and cluster 5 for OS and EFS. Cox proportional hazard model for multivariable analyses of cluster 1 and cluster 5 for OS and EFS. Abbreviations: HR, hazard ratio; CI, confidence interval; α CEBPAdouble−mutation status versus CEBPAwt, β FLT3ITD versus no FLT3ITD mutation, β NPM1mutant versus NPM1wt , δ WBC count higher than 20 × 109/L versus lower than 20 × 109/L, $ Age is used as continuous variable. [file 1471-2105-16-S4-S4-S5.pdf]

Additional file 5.

Variables	*P* -value	**HR**	95% CI-low	CI-high
Overall survival				
Cluster 1	**0.00617**	1.73	1.17	2.56
High cytogenetic risk	0.01237	1.80	1.14	2.86
Low cytogenetic risk	0.43231	1.17	0.79	1.74
FLT3 $^{ITD\ \beta}$	0.00005	1.91	1.40	2.61
NPM1 $^{+\ \beta}$	0.00178	0.59	0.42	0.82
WBC count$^{\delta}$, x10^9/L	0.00290	1.00	1.00	1.01
Age$^{\$}$	0.00009	1.02	1.01	1.03
Event-free survival				
Cluster 1	**0.00429**	1.77	1.20	2.62
High cytogenetic risk	0.00777	1.82	1.17	2.84
Low cytogenetic risk	0.41608	1.17	0.80	1.71
FLT3 $^{ITD\ \beta}$	0.00010	1.87	1.36	2.56
NPM1 $^{+\ \beta}$	0.00065	0.57	0.41	0.79
WBC count$^{\delta}$, x10^9/L	0.00480	1.00	1.00	1.00
Age$^{\$}$	0.00517	1.01	1.00	1.02
Overall survival				
Cluster 5	**0.02728**	1.49	1.05	2.14
High cytogenetic risk	0.03051	1.66	1.05	2.64
Low cytogenetic risk	0.67508	1.09	0.73	1.62
FLT3 $^{ITD\ \beta}$	0.00005	1.92	1.40	2.63
NPM1 $^{+\ \beta}$	0.00232	0.60	0.43	0.83
WBC count$^{\delta}$, x10^9/L	0.00656	1.00	1.00	1.01
Age$^{\$}$	0.00006	1.02	1.01	1.03
Event-free survival				
Cluster 5	**0.01886**	1.50	1.07	2.11
High cytogenetic risk	0.02308	1.67	1.07	2.58
Low cytogenetic risk	0.65032	1.09	0.75	1.60
FLT3 $^{ITD\ \beta}$	0.00017	1.83	1.33	2.50
NPM1 $^{+\ \beta}$	0.00142	0.59	0.42	0.81
WBC count$^{\delta}$, x10^9/L	0.00980	1.00	1.00	1.00
Age$^{\$}$	0.00593	1.01	1.00	1.02
