# Supplementary material for: An integrated approach of gene expression and DNA-methylation profiles of WNT signaling genes uncovers novel prognostic markers in Acute Myeloid Leukemia
Source: BMC Bioinformatics. 2015 Feb 23;16(Suppl 4):S4. doi: 10.1186/1471-2105-16-S4-S4 (PMC4347618; doi:10.1186/1471-2105-16-S4-S4)
Supplement: Additional file 6 — Venn diagram illustrating the overlap of prognostic markers detected by the integrative approach, the use of solely gene expression profiles, and solely DNA-methylation profiles. Using the integrative approach, seven prognostic markers are detected. Using solely gene expression profiles, 9 prognostic markers are detected. Using solely DNA-methylation profiles, 14 prognostic markers are detected. [file 1471-2105-16-S4-S4-S6.pdf]

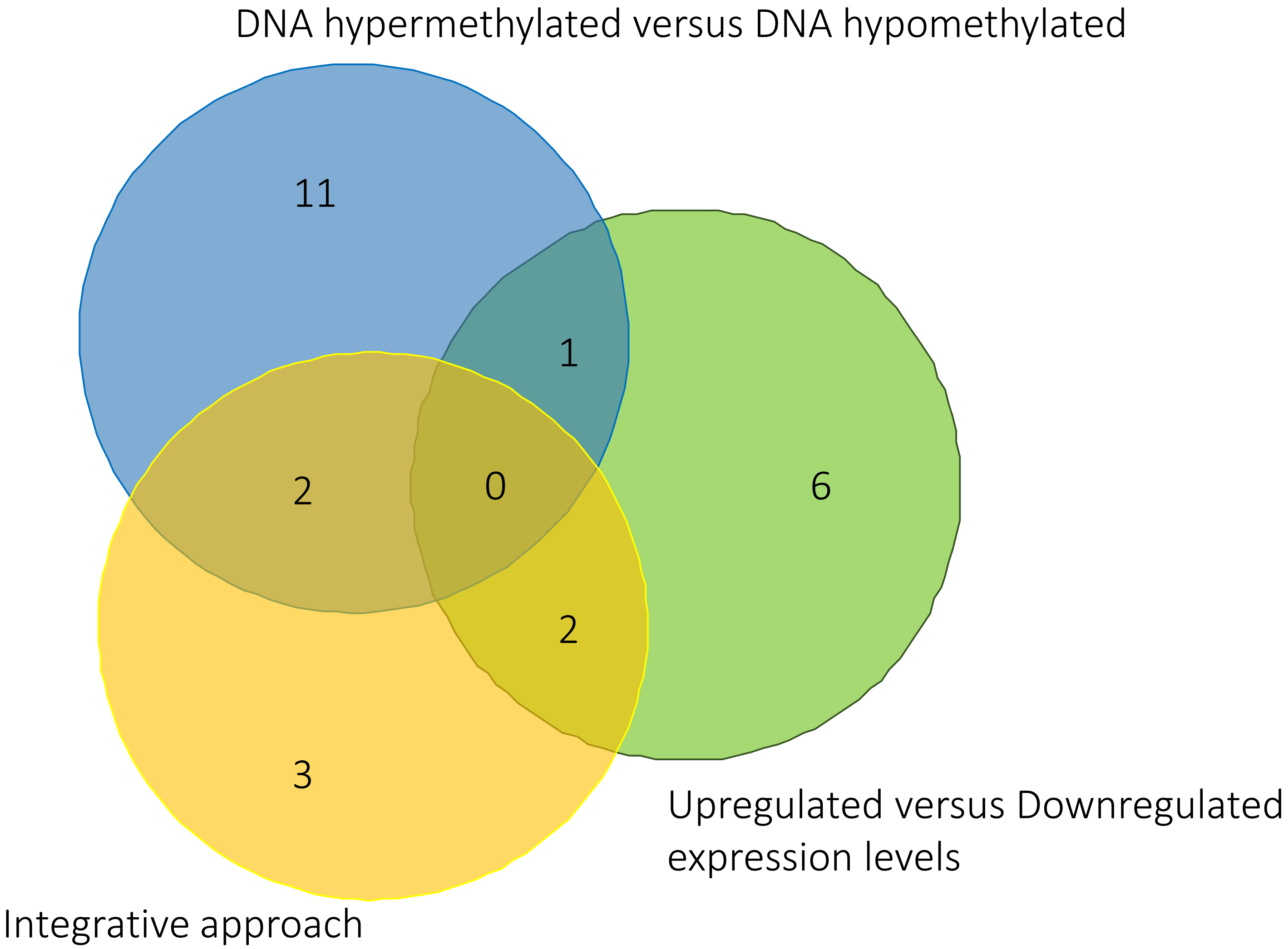
DNA hypermethylated versus DNA hypomethylated
11
1
2
0
6
2
3
Upregulated versus Downregulated expression levels
Integrative approach
